# Enzyme-Integrated Hydrogels for Advanced Biological Applications

**DOI:** 10.1021/polymscitech.5c00076

**Published:** 2025-07-31

**Authors:** Min Hu, Yujing Tang, Xingyue He, Kaohua Liu, Luping Qin, Xia Wang, Qigang Wang

**Affiliations:** † School of Pharmaceutical Sciences, 70571 Zhejiang Chinese Medical University, Hangzhou 310053, China; ‡ School of Chemical Science and Engineering, 12476 Tongji University, Shanghai 200092, China

**Keywords:** Enzyme-integrated hydrogel, Biocompatibility, Immobilization techniques, Biocatalysis, Biomedical diagnostics

## Abstract

Natural enzymes, with their precise structural organization and compartmentalization in metabolic pathways, exhibit remarkable catalytic efficiency, inspiring the design of enzyme-integrated hydrogels for advanced biocatalysis. In this review, we first systematically discuss commonly employed physical strategies—including self-assembly, electrostatic adsorption, and direct encapsulation—for constructing enzyme-integrated hydrogel systems. Subsequently, we elaborate on enzyme-mediated self-confined polymerization and cross-linking strategies, encompassing natural enzymes, enzyme cascades, nanozymes, and physical–biochemical coupling catalysis. When considering advanced biological applications, we highlight the diverse applications of enzyme-integrated hydrogels across various fields, including imaging, tumor therapy, tissue engineering, and other biomedical diagnostics and therapeutics. Lastly, we present critical perspectives on current research challenges and future opportunities for enzyme-integrated hydrogels, aiming to advance their development and expand their biomedical and clinical applications.

## Graphical abstract

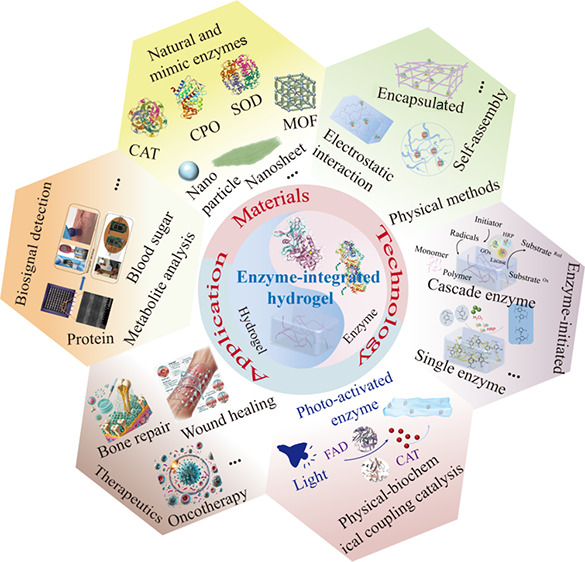

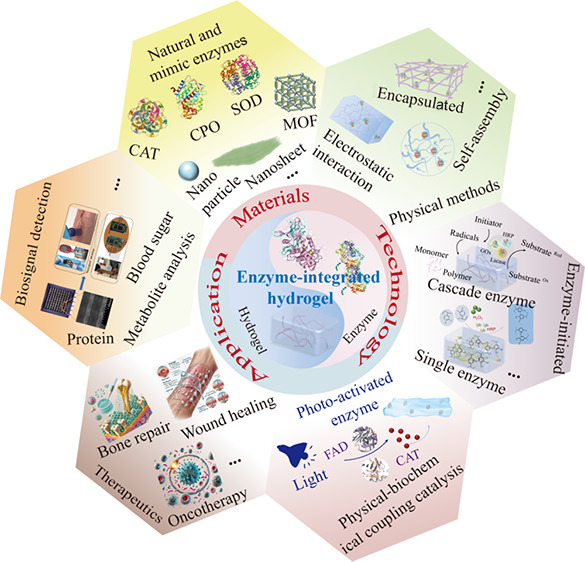

## 1 Introduction

Enzymes, whether proteins or RNA, play a vital role in cellular metabolism by regulating the catalytic processes involved in metabolism, energy transfer, and immune defense. These enzymes function as biocatalysts, orchestrating the intricate metabolic pathways of substances within living organisms.^1–4^ For efficient enzymatic reactions, a suitable medium (typically water) and moderate reaction conditions, such as optimal pH and temperature, are essential to ensure normal physiological functions.^4,5^ Unfavorable conditions, such as extreme temperatures and inappropriate pH levels, can significantly reduce enzyme efficiency, leading to abnormal catalytic reactions, disturbances in metabolic processes, and the initiation of diseases.^6–8^ Consequently, there is a strong correlation between biomedicine and the maintenance of enzyme activity. Researchers are increasingly interested in studying bioactive and eco-friendly materials for biomedical applications to transfer the advantageous properties of enzymes to in vitro systems.^9–11^ Recent advancements in recombinant DNA technology have enabled the large-scale production of various empirically validated enzymes. However, challenges remain because enzymes often exhibit reduced activity outside of cells, hindering their ability to perform effective and sustained reactions.^12,13^ Immobilized enzyme technology, which is central to modern biocatalysis, allows for flexible substrate selection and controlled reaction conditions. By immobilizing enzymes or enzyme combinations in specific areas, this technology facilitates effective, continuous, and recoverable biocatalytic processes.^14–18^ Additionally, immobilized enzyme technology offers significant advantages, including high enzyme activity, recyclability, control over the catalytic process, and prevention of enzyme contamination.^18,19^ The carrier materials used for immobilized enzymes must meet stringent criteria to ensure the activity and stability of various enzymes. For medicinal applications, these materials must possess excellent physicochemical features, such as prominent biocompatibility, mechanical strength, mass transfer efficiency, and stability.^20,21^

Hydrogels, characterized by their fully hydrated, cross-linked, 3D polymer networks, possess a range of distinctive attributes, including exceptional elasticity, notable stretchability, softness, elevated porosity, and insolubility. These unique properties render hydrogels increasingly indispensable in numerous contemporary biomedical applications, such as tissue engineering, deployment of antimicrobial agents, bioprinting, targeted drug delivery systems, wound healing, and the development of sensitive biosensors.^22–26^ Hydrogels with an extracellular matrix (ECM)-like structure can introduce exogenous entities into the body without causing damage while ensuring efficient mass transfer and high diffusion rates. Thus, they are preferred candidates for the immobilization of enzymes and multienzyme systems, promoting the construction of enzyme-integrated hydrogel platforms.^27–30^

Considering the emerging advancements in synthesis strategies, enzyme-integrated hydrogel techniques, which are increasingly being rationally designed, are now being applied to immobilize single, cascade, and mimetic enzymes. The primary objective of these techniques is to create an environment that closely resembles physiological conditions, safeguarding against denaturation and contamination. Furthermore, accumulating evidence indicates that enzyme-integrated hydrogel biomaterials have led to promising advances in the diagnosis and treatment of diseases such as cancer as well as tissue engineering and biosensing. In this review, the fabrication and development of bioactive enzyme-integrated hydrogels for biomedical applications are discussed (Figure 1). We begin by summarizing the physical methods for fabricating enzyme-integrated hydrogels such as encapsulating enzymes into the framework, utilizing electrostatic interactions, and promoting self-assembly. Next, we review the fabrication methods of enzyme-initiated self-confined polymerization and cross-linking in enzyme-integrated hydrogels, including single enzyme initiation, cascade enzyme initiation, mimic enzyme initiation, and physical–biochemical coupling catalysis, along with detailed mechanisms underlying the polymerization and cross-linking processes. Finally, we address the current challenges and prospects for optimizing the performance of enzyme-integrated hydrogels to enhance their therapeutic efficiency and broaden their clinical applications.

**Figure 1:**
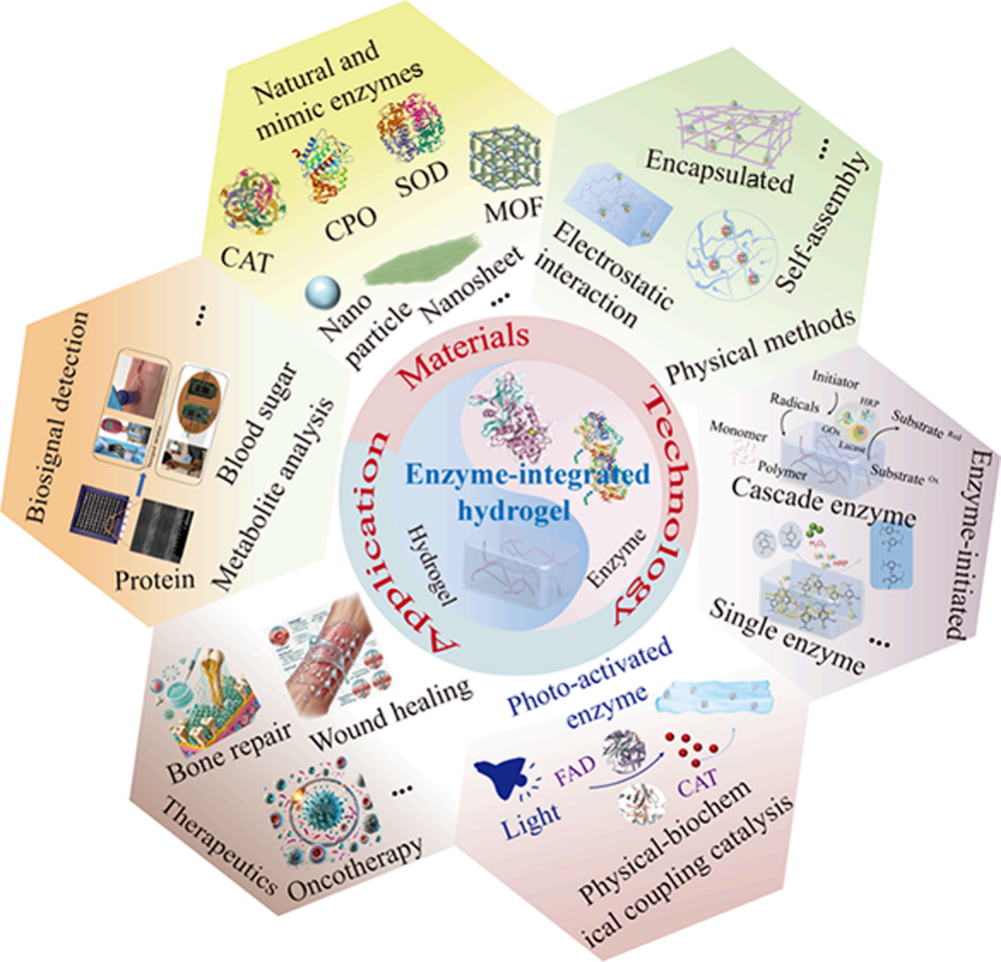
Schematic diagram illustrating the focus of this review on the fabrication of enzyme-integrated hydrogels and their biomedical applications.

## 2 Strategies for the Preparation of Active Enzyme-Integrated Hydrogels

The construction of compartmentalized systems leveraging biomimetic cellular structures to enhance the efficiency of biochemical reactions has attracted considerable attention in recent years.^31–37^ These confined systems offer multiple advantages. First, confined structures provide enzymes with a precisely controlled physicochemical microenvironment, characterized by optimized pH values, ion concentrations, and hydrophobic or hydrophilic properties, thus ensuring favorable catalytic conditions.^38,39^ Second, compartmentalization facilitates the establishment of catalytic pathways or substrate channels analogous to those in natural enzyme complexes, allowing substrates and products to traverse enzyme active sites more efficiently while minimizing undesirable side reactions.^40,41^ Third, such systems can reduce nonspecific interactions among enzymes, such as aggregation or denaturation, thereby significantly improving enzymatic stability and extending their functional lifespan.^42,43^ Additionally, these organized systems allow for dynamic regulation of reaction conditions through integration with functional materials, including porous hydrogels or nanogels.^44,45^ This synergy equips confined enzyme-hydrogel systems with adaptability to complex environmental conditions, broadening their applicability in diverse domains such as tissue engineering, disease diagnosis, and therapeutic interventions.

### 2.1 Enzyme-Integrated Hydrogels Fabricated by Physical Methods

Enzyme-integrated hydrogels fabricated via physical methods leverage interactions between enzyme surface properties—such as polar groups and apolar surface areas—and complementary hydrogel characteristics while simultaneously generating pores of suitable dimensions for enzyme encapsulation. This design ensures effective enzyme confinement while permitting free diffusion of substrates. Furthermore, self-assembly, electrostatic adsorption, and direct encapsulation techniques bypass the need for complex chemical processes or stringent environmental controls, enabling straightforward operation under mild conditions and enhancing enzyme storage stability, chemical resistance, and reproducibility. Among these, electrostatic adsorption exploits the electrostatic attraction between enzymes and the hydrogel matrix for immobilization via non-covalent interactions. Self-assembly utilizes precursor molecules (e.g., amphiphilic molecules, peptides) that spontaneously form dynamic networks through non-covalent interactions (hydrogen bonding, hydrophobic interactions, π–π stacking, and van der Waals forces), spatially confining enzymes within the three-dimensional network primarily through physical barrier effects. Direct entrapment/encapsulation relies on forming a network via physical or mild chemical cross-linking, mechanically immobilizing enzymes within interstitial spaces where spatial hindrance predominantly prevents diffusion and leaching (Figure 2). The following sections detail the principles, implementation, and specific advantages of these core physical fabrication approaches.

**Figure 2:**
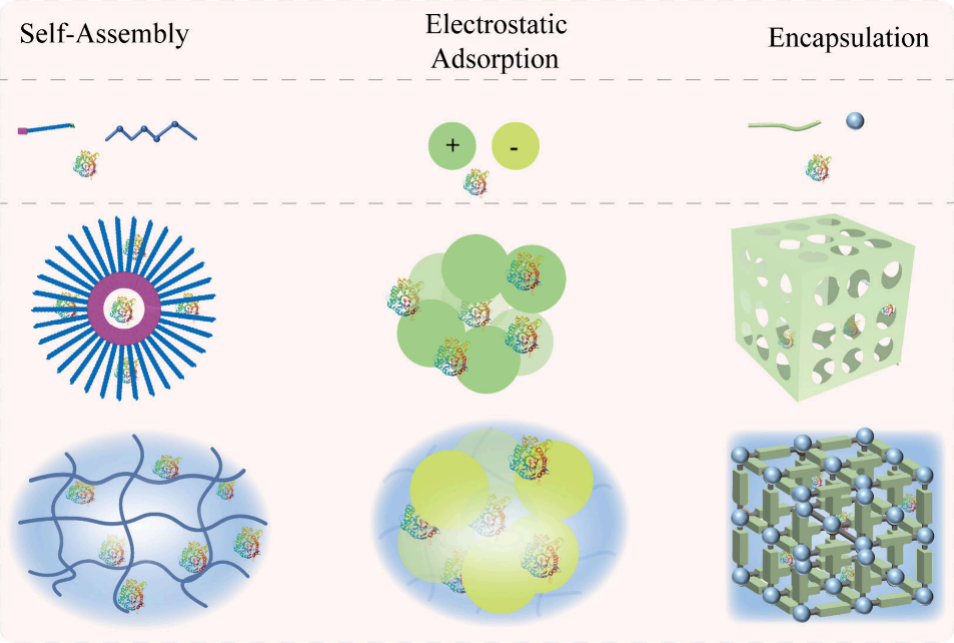
Enzyme-integrated hydrogels obtained through physical methods. (a) Schematic of enzyme-mediated self-assembly. (b) Absorption of enzymes onto the surface of the material by electrostatic interactions. (c) Encapsulation of the enzyme directly in the nanoparticle’s cavity or within a metal–organic framework.

#### 2.1.1 Self-Assembly

Molecular self-assembly is a ubiquitous process in biology, underpinning the formation of various complex biological structures, such as cell membranes, DNA, proteins, and cytoskeletons. It involves the spontaneous association of molecules under conditions close to thermodynamic equilibrium, where disordered individual molecules are linked by non-covalent interactions into stable, organized, and structurally well-defined aggregates.^46,47^ The concept of enzyme-assisted self-assembly (EASA) of low-molecular-weight hydrogelators, introduced by Xu, offers a novel method for obtaining hydrogels.^48^ The gelation process involves bond breaking rather than bond formation, regulating the balance of hydrophobicity and hydrophilicity of the amino acid amphiphilic derivatives to yield a hydrogelator. Specifically, alkaline phosphatase (ALP) is utilized to dephosphorylate the PO_4_^3–^ of *N*-(fluorenylmethoxycarbonyl) (Fmoc) tyrosine phosphate under mild conditions, altering the hydrophobicity of the precursor to trigger self-assembly. Xu et al. synthesized precursors of hydrogelators consisting of d-amino residues and l-amino acid residues, and by enzymatically dephosphorylating the enantiomeric substrates, they found that the removed phosphate was derived from tyrosine phosphate residues and that the chirality of the hydrogel precursors had a minimal effect on enzymatic hydrogelation. This work introduced a new class of molecular platforms for the generation of supramolecular hydrogels (Figure 3a).^49^ Since then, the preparation of hydrogels through ALP dephosphorylation to modify the hydrophilicity of peptide/protein sequences has garnered increasing research attention. For instance, Yang’s group employed hydrophobic short peptides coassembled with ovalbumin in the presence of phosphatase to create supramolecular hydrogels, which activated humoral immune responses and demonstrated potential applications as vaccine adjuvants.^50^ Chemiluminescent hydrogels were fabricated by coincubating the hydrogelator precursor Fmoc-Phe-Phe-Tyr­(H_2_PO_3_)-OH and the chemiluminescence agent AMPPD with ALP.^51^ Additionally, Tabata et al. designed a phosphatase-responsive PAAM hydrogel for stem cell culture due to its capacity to modulate the gene expression level of Runt-related transcription factor 2 in the presence of ALP.^52^ Small peptides produced through ALP dephosphorylation assemble in the silk fibroin solution to form stable hydrogels. Within these hydrogels, phosphate ions give rise to glycerophosphate, catalyzing the formation of calcium phosphate minerals within the porous structure. This process promotes cellular osteogenic differentiation and contributes to defect regeneration.^53^ Following the seminal work of Xu, EASA was expanded to other enzymes. For example, α-chymotrypsin has been applied to convert the water-soluble dipeptide ethyl ester “lego” (KL-OEt (K = lysine; L = leucine)) into oligopeptides, leading to a sol–gel transition.^54,55^ Matrix metalloproteinases (MMPs), enzymes overexpressed and secreted by various cancer cells, are extensively utilized in the construction of molecular self-assembled hydrogel integrases. Maruyama and colleagues reported a supramolecular gelator capable of inhibiting cancer cell growth by triggering molecular self-assembly in the presence of MMP-7 (Figure 3b).^56^ Haam and his colleagues developed an MMP-9-specific activatable peptide-probe-coupled fluorescent PEGDAAm-*co*-PAA hydrogel sensor.^57^ Furthermore, Hirst et al. used the enzyme subtilisin as a biocatalyst to catalyze hydrolysis, promoting the self-assembly, nucleation, and structural growth of Fmoc-dipeptide methyl esters to prepare supramolecular hydrogels.^58^ They investigated the impact of different enzyme concentrations on the fibrous supramolecule structure (Figure 3c) revealing that the catalytic activity and mobility of the biocatalytic clusters play a crucial role in determining the observation of induced supramolecular order. This work elucidates the critical role of engineered catalytic particles in the molecular self-assembly of next-generation soft nanomaterials and devices.

**Figure 3:**
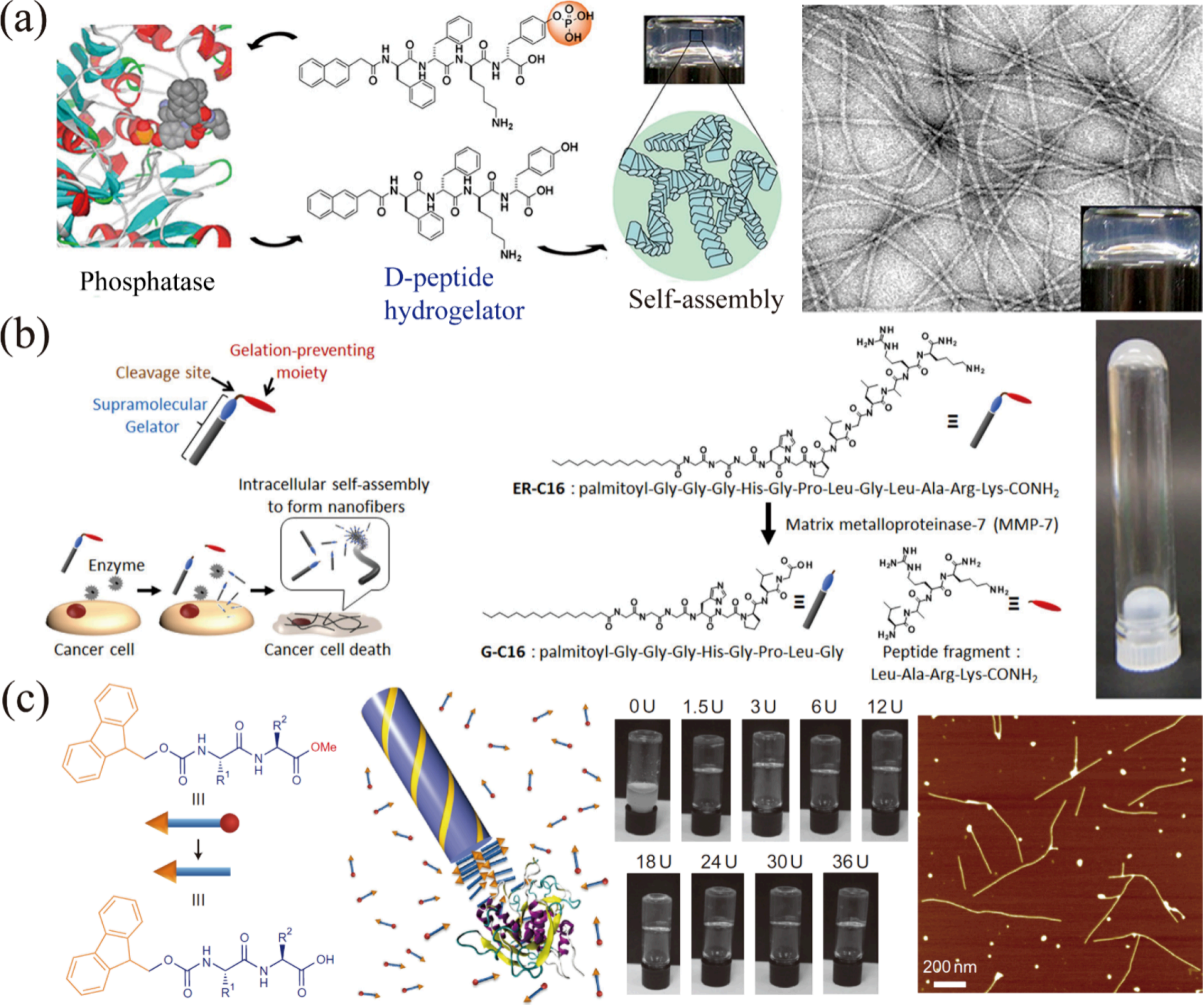
Enzyme-responsive molecular self-assembly toward supramolecular enzyme-integrated hydrogels. (a) Phosphatase enzymatic phosphorylation promotes the formation of d-peptide nanofibers. Reproduced from ref 49. Copyright 2013 American Chemical Society. (b) Schematic of the MMP-7 catalytic self-assembly process, illustrating the chemical structure of the precursor (ER-C16) and its corresponding gelator (G-C16). Reproduced from ref 56. Copyright 2014 American Chemical Society. (c) Enzymatic hydrolysis facilitates the self-assembly, nucleation, and structural growth of Fmoc-dipeptide methyl ester. Reproduced with permission from ref 58. Copyright 2010 Springer Nature.

The utilization of enzyme-mediated self-assembly for hydrogel formation offers distinct advantages over conventional methods, such as heating–cooling, pH adjustment, and ultrasonic treatment. Enzyme-mediated self-assembly presents a milder yet more efficient alternative, facilitating hydrogel formation at specific locations, which is particularly valuable for applications in disease diagnosis and treatment. Currently, the most widely used enzymes employed for hydrogel preparation through enzyme-controlled self-assembly are ALP and MMPs. Further research is warranted to identify additional enzymes with enhanced properties tailored to specific applications.

#### 2.1.2 Electrostatic Adsorption

Electrostatic immobilization has emerged as a significant research focus within biomaterials. This technique achieves enzyme immobilization through electrostatic attraction between oppositely charged enzyme molecules and the hydrogel network (or its components), integrating enzymes onto the hydrogel surfaces or within the internal pores. Compared to traditional covalent methods, this approach offers distinct advantages, including mild reaction conditions, reversible binding, and better preservation of the enzyme conformation. For instance, positively charged carriers like polyethylenimine (PEI)-modified substrates effectively capture negatively charged enzymes (e.g., acetylcholinesterase) via electrostatic attraction. Studies have shown that Fe_3_O_4_@MXene@PEI gel achieves an exceptionally high immobilization efficiency of 98.8% for acetylcholinesterase, primarily attributed to strong electrostatic interactions between the gel’s abundant amino groups and enzyme molecules.^59^ Similarly, Zhang et al. stabilized laccase on a p*K*_a_-matched cellulose hydrogel doped with β-cyclodextrin using charge-assisted hydrogen bonding, achieving a loading capacity of 754.5 mg g^–1^ while retaining over 80% of the initial activity.^60^ Hu et al. immobilized cellulase on a hydrogel cross-linked from cationic guar gum, anionic acrylamide, and dopamine, resulting in broader pH adaptability, improved storage stability, and high activity at elevated temperatures.^61^ Besides, polyacrylamide hydrogel microspheres synthesized via the reverse emulsion method can adsorb lipase through hydrogen bonding, creating a micro water environment to enhance the stability and activity of the enzyme.^62^

Electrostatic interactions, being weaker than covalent bonds, can act as sacrifical bonds. When the system experiences stress, these interactions preferentially break, dissipating energy throughout the network and contributing to hydrogel toughness. Gong’s research group demonstrated this by preparing polyampholyte hydrogels via random copolymerization of anionic monomers and cationic monomers.^63^ Within this network, strong ionic bonds act as permanent cross-links, while weaker, reversible ionic bonds dissipate energy, endowing the hydrogel with excellent mechanical properties and self-healing capabilities. For enzyme-integrated hydrogels, synergistic integration with other interactions can enhance enzyme–carrier binding strength, promoting efficient immobilization and long-term stability.

Despite progress, constructing enzyme-integrated hydrogels via electrostatic interactions faces persistent challenges. Foremost is long-term stability. In complex physiological environments, salt ions can shield electrostatic interactions, leading to gradual enzyme leaching. Strategies employing synergistic multiforce interactions offer a promising solution. Examples include utilizing catechol–metal coordination bonds to enhance adhesion under physiological saline conditions and developing dynamic covalent bonds to improve the overall stability. Additionally, precise control over enzyme orientation is crucial for enhancing the activity of immobilized enzymes. Traditional electrostatic immobilization often results in random adsorption, potentially obscuring or distorting active sites. Further research should therefore explore site-specific immobilization guided by molecular simulations, such as computational modeling to predict enzyme surface charge distribution and designing specific binding peptide sequences for optimal orientation.

#### 2.1.3 Direct Encapsulation Approaches

Direct encapsulation involves incorporating enzymes into polymer networks using methods such as gel/fiber embedding, entrapment, encapsulation, and metal–organic framework (MOF) embedding.^64,65^ A multifunctional enzyme immobilization platform was developed using PtCu hydrogel coated with a layer of an amorphous zeolitic imidazolate framework (ZIF-8) (Figure 4a). The PtCu hydrogel exhibits peroxidase activity and enhances enzyme activity by continuously supplying metal ions through the proximity effect, increasing enzyme activity to approximately 2.4 times that of the free enzyme. The MOF shell protects the internal enzyme from inactivation caused by harsh environments.^66^ Additionally, Tan and colleagues devised a strategy to prepare multistage microporous and mesoporous ZIFs for encapsulating glucose oxidase (GOx) and horseradish peroxidase (HRP) using hydrogels as templates, thereby avoiding the encapsulation hindrance associated with small pores of MOFs. This method resulted in a 7.7-fold enhancement in activity, significantly surpassing that of free enzymes and exhibiting a 2.7-fold increase compared to those adsorbed on conventional microporous MOFs.^67^ Furthermore, a MOF nanoenzyme (PEG@Zr-Fc) hydrogel system has been reported for immobilizing mimetic enzymes, preventing potential toxicity problems associated with the release of mimetic enzyme (Figure 4b).^68^ Another study explored immobilizing lipase on cellulose gel microspheres through physical adsorption, improving enzyme activity and stability.^69^ Additional research introduced an approach to enzyme immobilization by leveraging the affinity between metals and proteins, along with the unique porous structure of nanozyme hydrogels, simplifying the immobilization process and significantly enhancing peroxidase activity, reproducibility, and stability.^70^

**Figure 4:**
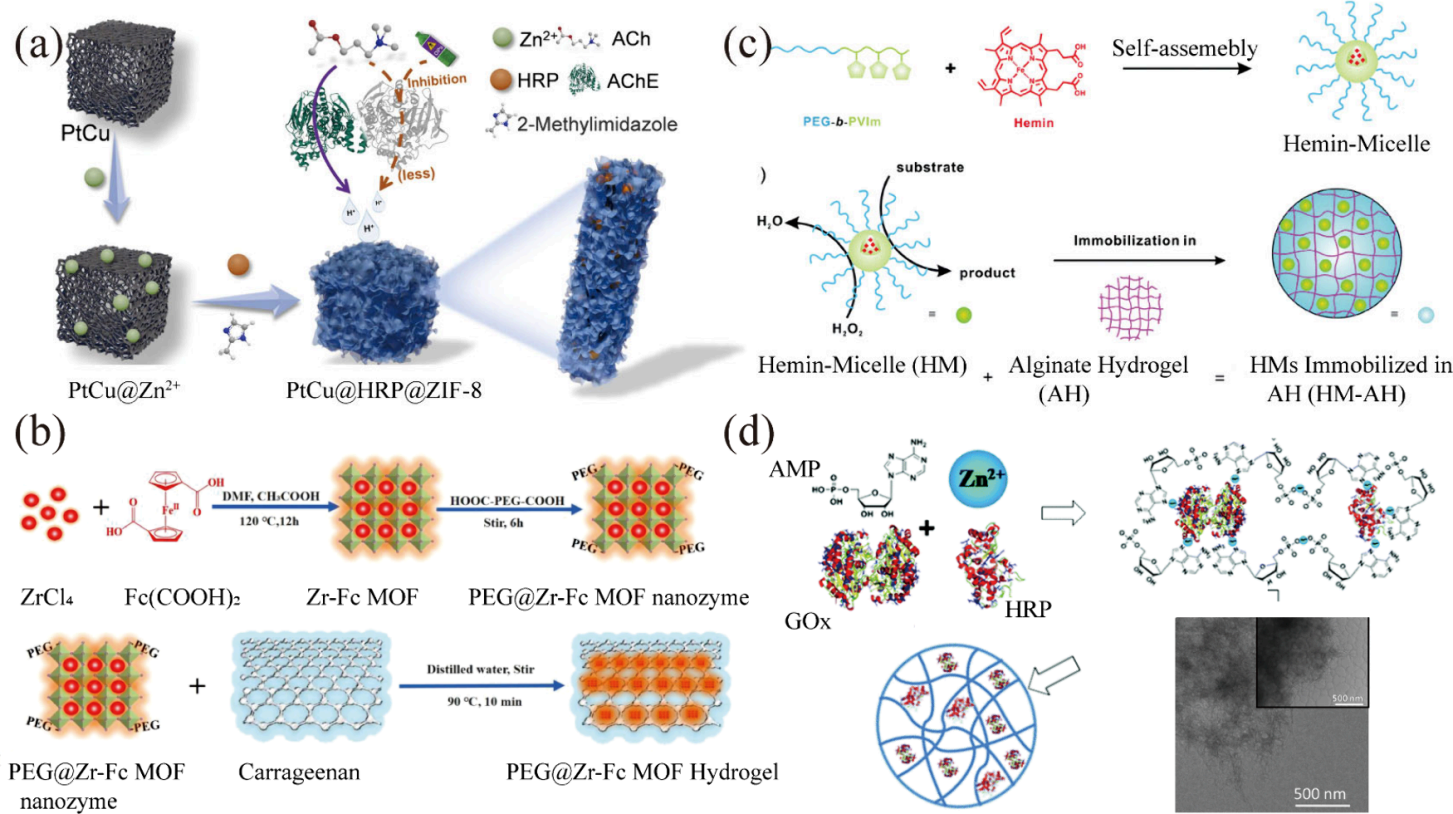
Enzyme-integrated hydrogel was fabricated via a direct encapsulation method. (a) HRP was immobilized in a PtCu hydrogel coated with amorphous MOFs. Reproduced with permission from ref 66. Copyright 2022 Elsevier. (b) A HCOOH-PEG-COOH modified Zr-MOF nanozyme was mixed with carrageenan to construct a simulated enzyme-integrated hydrogel system. Reproduced with permission from ref 68. Copyright 2021 Acta Materialia. (c) Block copolymers were immobilized in a supramolecular alginate hydrogel after coassembly with hemin. Reproduced with permission from ref 71. Copyright 2017 Royal Society of Chemistry. (d) Different protease species were immobilized into nanofibrils through the coordination of zinc ions with AMP. Reproduced with permission from ref 72. Copyright 2016 Royal Society of Chemistry.

The self-assembly entrapment of enzymes is an appealing strategy due to its high operational stability and minimal alterations in the enzyme structure. For instance, Yacoby and colleagues utilized self-assembly of peptides to encapsulate the [FeFe]-hydrogenase enzyme, effectively limiting oxygen diffusion and penetration while preserving enzyme conformation and activity.^27^ Another method involves preparing mimetic enzyme micelles by coassembling hemin with block copolymers (PEG-*b*-PVIm), which are then immobilized within alginate hydrogels (Figure 4c). The hemin micelles serve as the catalytic center, while the hydrogel enhances catalytic activity and stability and functions as a recognition center with substrate selectivity.^71^ This study introduces a straightforward approach for coimmobilizing multiple enzymes within metal-coordinated hydrogel nanofibers. Various proteases were immobilized through the self-assembly of adenosine monophosphate (AMP) and zinc ions, resulting in fiber formation in an aqueous medium (Figure 4d). Notably, the immobilized enzymes exhibited enhanced stability under harsh conditions, including high temperatures, extreme pH levels, and organic solvents, retaining 70% of their original activity.^72^ Additionally, extensive research into incorporating enzymes into composite systems has demonstrated advantages such as improved thermal resistance, enhanced stability, facilitated recovery, and activity over a wide pH range, paving the way for diverse applications in various fields.^73–75^

In summary, physical methods—including self-assembly, direct wrapping, and electrostatic adsorption—provide effective strategies for constructing enzyme-integrated hydrogels. These approaches feature relatively simple operation, mild conditions, and maximal retention of the enzyme activity. Their core principle relies on ingeniously leveraging non-covalent interactions—such as hydrophobic forces, hydrogen bonding, steric hindrance, and electrostatic attraction—to achieve gentle enzyme entrapment within the hydrogel’s three-dimensional network. Selecting an appropriate method requires careful consideration of application-specific requirements for enzyme loading capacity, stability, release kinetics, responsiveness, and operational simplicity (see Table 1). Despite persistent challenges in preventing enzyme leakage and ensuring long-term stability, the inherent mildness and reversibility of these physical strategies establish crucial foundations for designing stimuli-responsive, reusable enzyme delivery systems. Future research will focus on optimizing network structures to precisely control the enzyme spatial distribution and microenvironment, developing novel stimuli-responsive physical materials, and designing composite immobilization strategies using synergistic multiple physical interactions, all aimed at enhancing the performance and application potential of physically immobilized enzyme-integrated hydrogels.

**Table 1:** Comparative Analysis of Physical Methods for Enzyme-Integrated Hydrogels

property	self-assembly	electrostatic adsorption	direct encapsulation
mechanism	spontaneous network formation via non-covalent interactions	adsorption of oppositely charged enzymes onto pre-formed hydrogels via Coulombic attraction	enzyme mixed with precursors prior to gelation
integration pathways	spatial confinement (dominant) + weak non-covalent interactions	electrostatic attraction	physical entrapment (steric hindrance)
operational simplicity	moderate (requires assembly optimization)	moderate (requires incubation)	high (single-step mixing/gelation)
enzyme loading capacity	moderate–high	low–moderate (limited by surface area/charge density)	high
enzyme distribution	throughout network	surface/internal pore walls	heterogeneous within matrix
enzyme leakage risk	moderate–high (due to dynamic networks)	moderate–high (environment-dependent)	high (typically exhibits significant burst release)
enzyme activity retention	high (mild assembly process)	high (no chemical modification)	moderate (harsh gelation microenvironments)
reversibility/controlled release	high (stimuli-responsive networks)	high (modulated by pH/ionic strength)	low–moderate (dependent on degradation/diffusion)
applicable enzyme types	broad (assembly-compatible)	charge-dependent (pH-specific)	broad
exemplary materials	self-assembling peptides, amphiphilic polymers	chitosan (+), alginate (−), poly(acrylic acid) (−)	alginate (Ca^2+^-cross-linked), gelatin, pNIPAM
key advantages	dynamic responsiveness, injectability, biomimetic properties	enzyme replaceability, surface functionalization flexibility	operational simplicity, high loading capacity, low cost
major limitations	low network stability, leakage vulnerability	low loading capacity, strong environmental sensitivity	low network stability, leakage vulnerability

### 2.2 Enzyme-Initiated Polymerization and Cross-Linking for the Construction of Enzyme-Integrated Hydrogels

Compared to physical methods for constructing enzyme-integrated hydrogels, chemical immobilization via enzyme-initiated polymerization and cross-linking offers significant advantages. This approach not only preserves the native conformation and activity of enzymes while protecting them from adverse external factors but also facilitates the diffusion of substrates and products, enhances catalytic efficiency, enables the construction of multienzyme systems, and allows for flexible functionalization designs tailored to specific application requirements.^76–78^

Traditional monomer polymerization and cross-linking processes often involve harsh conditions, such as intense light, high temperature, or aggressive chemical reactions. In contrast, enzymes provide an attractive alternative due to their highly efficient catalytic properties under mild and controllable reaction conditions. Enzyme-initiated polymerization and cross-linking leverage these inherent enzymatic traits, resulting in high specificity and purity of the final polymeric product. Moreover, this enzymatic approach facilitates the formation of compartmentalized architectures and confined gel networks that effectively mimic natural cellular structures. Such biomimetic configurations significantly enhance the catalytic efficiency and promote the transport of bioactive substances. A representative example is an enzymatic cascade system involving oxidase and peroxidase: the oxidase first oxidizes its substrate to generate hydrogen peroxide. Subsequently, in the presence of peroxidase, this H_2_O_2_, along with oxygen, oxidizes an initiator to produce free radicals. These radicals then initiate the polymerization and cross-linking reactions, ultimately constructing the three-dimensional hydrogel network (Figure 5).

**Figure 5:**
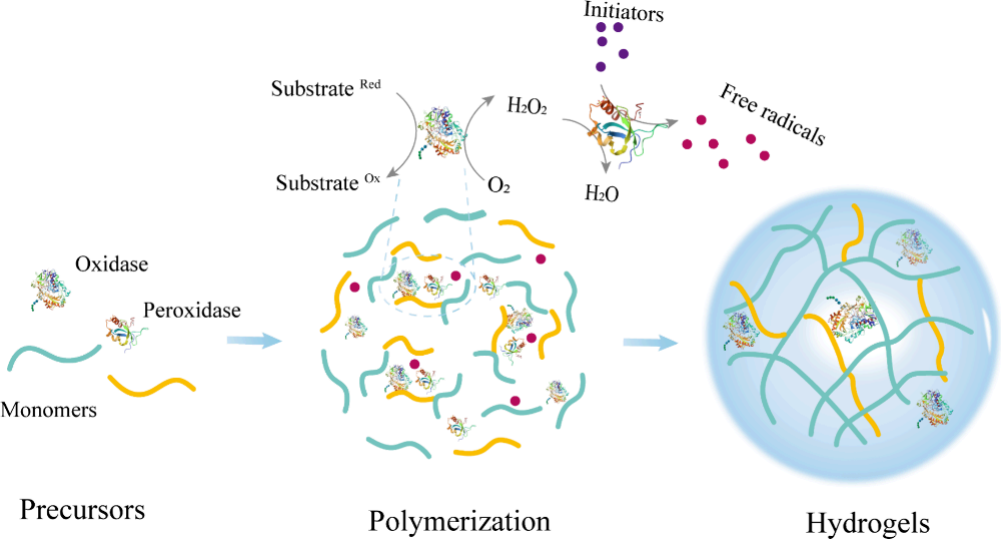
Enzyme-integrated hydrogels were fabricated by an oxidase and peroxidase cascade system.

Consequently, enzyme-initiated polymerization and cross-linking represent not only a method for producing purified polymeric materials but also one of the mildest and most attractive techniques for constructing enzyme-integrated hydrogels. The versatility and efficacy of this approach are further demonstrated by the successful utilization of various enzymes, including HRP, laccase, tyrosinase (TYR), transglutaminase (TGase), lysyl oxidase (LOX), and plasma amine oxidase (PAO), for polymerization and cross-linking in hydrogel formation.^79–84^

#### 2.2.1 Single-Enzyme-Mediated Catalysis Systems

The specific cross-linking mechanism of certain enzymes is depicted in Figure 6. As a prototype of multinuclear copper-containing oxidases, laccase catalyzes the oxidation of various aromatic substrates, such as anilines, polyphenols, methoxy-substituted phenols, and other compounds, by directly reducing molecular oxygen in the solvent.^85^ This process yields an unstable reactive aromatic radical, which subsequently undergoes coupling to form different covalent bonds (Figure 6a). The most common enzyme in the peroxidase family is HRP, a low-redox-potential heme peroxidase that catalyzes the coupling of phenol, aniline, and their derivatives in the presence of hydrogen peroxide (Figure 6b).

**Figure 6:**
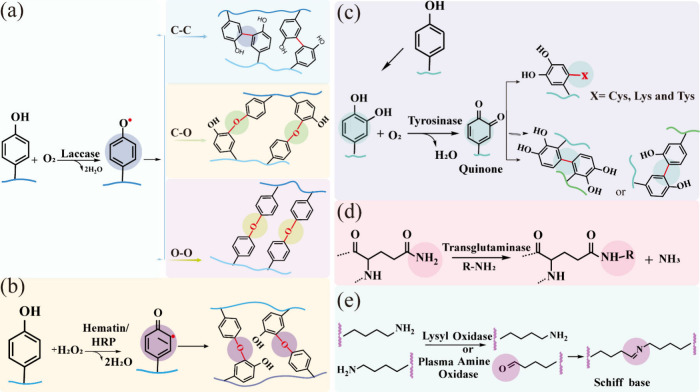
Mechanism of single-enzyme-mediated catalytic polymerization and cross-linking: (a) laccase; (b) hematin/HRP; (c) tyrosinase; (d) transglutaminase; (e) lysyl oxidase or plasma amine oxidase.

Utilizing laccase’s ability to oxidize catechol and establish a C–N bond through Michael addition or radical coupling with free amino groups of chitosan in the early stages,^86,87^ the Guebitz group used laccase-activated phenolics as cross-linkers to create chitosan hydrogels, avoiding the use of toxic cross-linking agents and offering a new environmentally friendly approach.^88^ Additionally, the literature documents that laccase catalyzes natural compounds like polysaccharides and peptides to create hydrogels for biological applications.^89,90^ Gelation is achieved through the oxidation of catechol moieties on the poly­(citric acid-*co*-poly­(ethylene glycol))-*g*-dopamine (PCPD) chain, facilitated by HRP and H_2_O_2_-generated oxygen radicals (Figure 7a). This oxidation process leads to either the cross-linking of catechol moieties or their reaction with the amino group of amino-modified PF127, resulting in the formation of hydrogels with rapid gelation, high mechanical strength, resistance to swelling, and degradability.^91^ Furthermore, a poly­(γ-glutamic acid) hydrogel with enhanced tissue adhesive properties was successfully fabricated via HRP/H_2_O_2_ cross-linking of a catechol moiety (Figure 7b).^92^ pH-responsive hydrogels laden with drugs for oral administration can be effectively created using the same enzymatic cross-linking method.^93^ A range of in situ-forming hydrogels based on gelatin,^94^ hyaluronic acid (HA),^95^ dextran,^96^ and peptides^97^ were fabricated utilizing HRP/H_2_O_2_ to catalyze oxidative coupling of phenol moieties preconjugated on the polymer backbone. The gelation rate and mechanical properties of these hydrogels were shown to be modifiable independently through alternations in HRP and H_2_O_2_ concentrations.^98^ Despite these advances, concerns remain regarding the HRP/H_2_O_2_ system used for the catalytic synthesis of hydrogels. The use of H_2_O_2_ in the catalytic process can cause oxidative damage to the surrounding tissue. Additionally, the introduction of HRP into the body may trigger potential immunogenic reactions.^99^ To address these concerns, researchers have suggested minimizing the use of H_2_O_2_ to achieve gelation or employing hematin and its derivatives as peroxidase-like catalysts that lack HRP epitopes to avoid immunogenicity.^100^ For instance, Shen et al. designed a system comprising natural silk fibroin protein, glucose, GOx, and hemoglobin. Under the catalysis of hemoglobin, tyrosine radicals were generated, facilitating the construction of a tyrosine-cross-linked hydrogel. Notably, the tyrosine residues on silk fibroin in the hydrogel stabilized the hemoglobin structure during the oxidation process, imparting the system with heightened catalytic efficiency and sustained antioxidative properties (Figure 7c).^101^ Additionally, in situ HRP-free hydrogels have been reported, wherein HRP was immobilized on modified silica nanoparticles by reductive amination reaction using poly­(ethylene glycol) spacers, followed by catalytic cross-linking of polymer–phenol conjugates. These hydrogels not only had adjustable stiffness and gelation rates but also exhibited a low inflammatory response (Figure 7d).^102^

**Figure 7:**
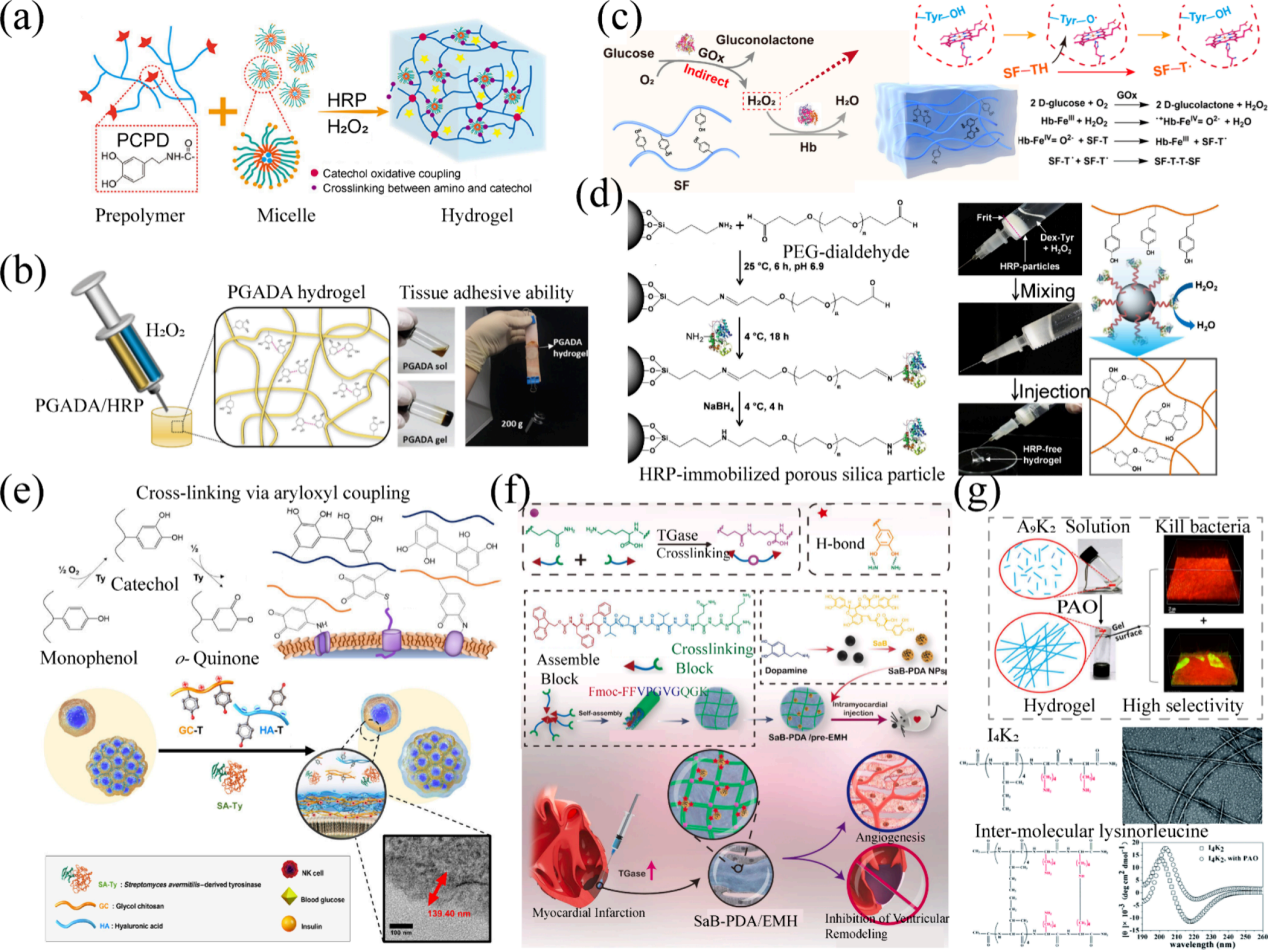
(a) Single-enzyme-mediated catalytic polymerization for the fabrication of enzyme-integrated hydrogels. Reproduced from ref 91. Copyright 2023 American Chemical Society. (b) Formation of the hydrogel network through single-enzyme-initiated oxidative polymerization and cross-linking of catechol using the HRP/H_2_O_2_ catalytic system. Reproduced from ref 92. Copyright 2020 American Chemical Society. (c) Utilization of hemoglobin as a peroxidase-like catalyst to generate tyrosine radicals and form silk fibroin hydrogel via cross-linking. Reproduced from ref 101. CC BY 4.0. (d) Synthesis of hydrogels with adjustable stiffness by immobilization of HRP onto modified silica nanoparticles via a reductive amination reaction and subsequent catalyzed cross-linking of polymer–phenol conjugates. Reproduced with permission from ref 102. Copyright 2018 Acta Materialia. (e) TYR-catalyzed oxidation of monophenol-modified ethylene glycol chitosan (GC) and hyaluronic acid (HA) to form quinone-containing polysaccharides that polymerize and cross-link on cell surfaces, forming a hydrogel nanofilm. Reproduced from ref 107. CC BY-NC 4.0. (f) Peptide sequences containing TGase cross-linking blocks enable the preparation of mechanically enhanced elastic protein-like hydrogels via TGase catalysis. Reproduced with permission from ref 112. Copyright 2021 Elsevier. (g) Amphiphilic peptides with lysine residues serve as substrate amino acids for PAO/LOX, enabling the oxidative cross-linking of lysine side chains to prepare antibacterial hydrogels. Reproduced from ref 116. Copyright 2016 American Chemical Society.

TYR, a copper-containing polyphenol oxidase widely distributed in nature, is employed for the oxidation of phenolics.^103^ This oxidation process involves two consecutive reactions: initially, TYR hydroxylates phenols by adding a hydroxyl group at the ortho position, forming catechol. Subsequently, catechol is further oxidized to generate quinones. These quinones readily produce free radical intermediates that are essential for cross-linking. Alternatively, active quinones form covalent bonds with thiols, amines, or other phenolic moieties through various chemical reactions, including Michael addition, the Maillard reaction, and oxidative phenol coupling (Figure 6b).^80,104,105^ Notably, TYR derived from *Streptomyces avermitilis* is extensively utilized due to its advantageous characteristics, such as a flat surface, broad substrate entrance, and short distance from the surface to the active site, facilitating the efficient oxidation of tyrosine residues on the surface of polysaccharides or proteins. It has been reported that sprayable adhesive hydrogels, with a gelation time of less than 50 s, were successfully synthesized through the site-specific coupling of tyramine-conjugated HA and gelatin (HG) using a novel TYR derived from *S. avermitilis*.^106^ Additionally, monophenol residues of glycol chitosan (GC-T) and HA, capable of enzymatic oxidation by TYR, were synthesized through the monophenolic modification of ethylene glycol chitosan (GC) and HA. The oxidized monophenols rapidly interacted with organic and inorganic substrates through hydrogen bonding, Schiff base reactions, and Michael addition, forming a multilayer hydrogel nanofilm on the cell surface that achieved long-term blood glucose regulation (Figure 7e).^107^

Transglutaminase (TGase) catalyzes the acyl-transfer process between the carboxylic acid groups of glutamine residues (acyl donors) and various primary amines (acyl acceptors), including the ε-amino groups of lysine residues in certain proteins (Figure 6d). Enzyme-loaded hydrogels, formed through TGase-mediated cross-linking reactions, offer exceptional biocompatibility and stability for biomedical applications, obviating the requirement for supplementary reagents.^108^ The literature indicates that intracellular TGase can facilitate the formation of nanoparticles from short peptides, while plasma TGase is employed to enhance the cross-linking and coagulation processes of short peptide fibers.^109,110^ This is due to the rapid conversion of coagulation factor XIII in the blood into activated TGase, which cross-links fibrin monomers, resisting fibrinolysis and proteolysis. As a result, there has been an increase in research on in situ injection molding/curing of hydrogels, leading to their expanded use in the biomedical field, particularly for hemostatic materials. Recently, an extraluminal arterial hemostatic hydrogel was developed for rapid blood loss control, demonstrating strong adhesive properties on moist biological tissue surfaces. This hydrogel is constructed through the Schiff base reaction between oxidized PEG (OPEG) and aminated gelatin (AG) as well as the TGase cross-linking reaction involving gelatin (AG) and glutamine (G), making it a viable sealant for vascular hemostasis.^111^ Additionally, it was through the catalytic cross-linking of TGase, highly expressed in the heart after myocardial infarction, that the mechanical strength of hydrogel was enhanced, as reported by Chen and colleagues. They designed a novel peptide sequence incorporating self-assembly blocks (Fmoc-FF), framework blocks (VPGVC), and TGase cross-linking block (QGK) to create an injectable hydrogel for intramyocardial injection. Through TGase enzyme-catalyzed reactions, they formed a network structure resembling cross-linked chains of elastic proteins, aiming to inhibit ventricular remodeling and promote angiogenesis (Figure 7f).^112^

Lysyl oxidase (LOX) and plasma amine oxidase (PAO) are copper-dependent amino oxidases that convert lysine and hydroxylysine residues in collagens and elastin into highly reactive aldehydes, which then combine with other oxidized groups or intact lysines to form various inter- and intrachain cross-links.^113,114^ Research has demonstrated that seeding MSCs into hydroxyapatite–poly­(lactide-*co*-glycolide) (HA-PLG) scaffolds, followed by LOX treatment, induces enzymatic collagen cross-linking. This process enhances osteoblast differentiation and improves the mechanical properties of engineering constructs.^115^ Additionally, amphiphilic peptides containing lysine residues can act as substrate amino acids for PAO/LOX, which catalyzes the oxidation of lysine side chain primary amines, leading to a sol–gel transition. The resulting hydrogel exhibits excellent antibacterial properties (Figure 7g).^116,117^ Combining radical polymerization and enzyme-induced cross-linking represents a new strategy for gelation. Specifically, single-network EPL-*g*-poly­(NVP-*co*-NMA) hydrogels can be synthesized by the radical polymerization of 1-vinyl-2-pyrrolidinone (NVP) and *N*-methylol acrylamide (NMA) in the presence of ε-poly-l-lysine (EPL). Subsequent addition of PAO oxidizes the primary amines of EPL, forming a secondary cross-linking network via the Schiff base reaction. These enzyme-induced dual-network hydrogels have demonstrated the capacity to accelerate wound healing efficiency and hold a significant potential in biomedical applications.^118^

Besides the aforementioned enzymes, an extensive range of enzymes, including sortase, phosphopantetheinyl transferase, P450, phosphatases, β-lactamase, and thermolysin, have been employed to facilitate the sol–gel conversion through distinct catalytic mechanisms. Nevertheless, the efficacy of a single enzyme in a free environment often encounters challenges in achieving rapid and efficient catalytic behavior, limiting its applications in biological contexts.^119,120^

#### 2.2.2 Cascade-Enzyme-Mediated Catalysis Systems

Inspired by the involvement of intracellular multienzyme/cascade enzyme systems in metabolic processes, the development of a cascade-enzyme-mediated hydrogel integrase system offers several advantages by providing a domain-limited microenvironment and a substrate diffusion channel, effectively overcoming the limitations inherent in single-enzyme catalysis. Articles that categorize cascade enzyme reactions (linear, parallel, orthogonal, cyclic, and triangular) based on the environments involved (intracellular or extracellular) and the number of participating enzymes have been extensively reported,^121^ so these concepts will not be further elaborated on here. In practical applications, such as drug and protein sequence syntheses, a variety of cascading strategies are typically employed to synthesize the desired product. Notably, linear and parallel cascading reactions are the primary methodologies for synthesizing enzyme-laden hydrogels in multienzyme cascading reactions. These methods offer significant advantages over those of other cascade systems and single-enzyme catalytic systems. Specifically, the linear cascade enzyme system allows the catalytic product of the first enzyme to serve directly as the substrate for the second enzyme, avoiding the generation of unstable reaction intermediates and the inefficient diffusion of enzyme intermediates, thereby enhancing the reactivity and catalytic efficiency. Additionally, parallel cascades between complementary enzymatic processes enable the reciprocal utilization of cofactors through complementary coupling. This significantly reduces the presence of unstable intermediate components, facilitates smoother transitions between reaction phases, and ultimately enhances the overall efficiency of the enzymatic reaction. Oxidases and peroxidases mediate linear cascading reactions, which are exemplary for constructing hydrogels due to the well-controlled consumption–production equilibrium in the linear cascading process (Figure 8a), effectively regulating the concentration of H_2_O_2_ used for initiating and inhibiting polymerization. In the linear reaction with HRP, various oxidase enzymes are involved, including GOx, galactose oxidase (GalOx),^122^ choline oxidase (ChOx),^123^ alcohol oxidase (AOx),^124^ cholesterol oxidase (COD),^125^ sarcosine oxidase (SOx),^126^ xanthine oxidase (XOx),^127^ amino acid oxidase (AAO),^128^ and lactate oxidase (LOx).^129^ For instance, Wang et al. developed composite hydrogels by initiating the immediate cross-linking of chondroitin sulfate grafted with tyrosine and a double bond (GMA-CS-Ph-OH) and the polymerization of monomers through the GOx/HRP cascade system (Figure 9a).^130^ The tyrosine-modified chondroitin facilitated the cross-linking, while the cascade catalyzed by GOx and HRP led to the formation of polymeric/cross-linked hydrogels. This process involved pre-cross-linking followed by in situ polymerization, resulting in composite hydrogels with improved toughness. Similarly, Strakosas et al. utilized a cascade system of oxidases and HRP to “grow” electrodes inside the brain.^131^ Specifically, an oxidase (GOx or LOx) and HRP, along with ETE-COONa, were embedded in a PVA:PLL polymer matrix. The preparation involved an amidation reaction with EDC/sulfo-NHS, which then reacted with ETE-COONa to produce the intermediate ETE-NHS ester. This ester further reacted with the primary amine of PLL, the hydroxyl group of PVA, and amino and alcohol groups in the tissue to construct the gel. Subsequently, the ROX and HRP cascade system, fueled by endogenous metabolites in neural tissue, catalyzed the production of polymerizable ETE radicals. The polymerized ETE-NHS cross-linked with PLL to form a stable pETE-PLL conductive polymer hydrogel electrode (Figure 9b).^131^ This innovative method leverages an endogenous metabolite to trigger enzyme activity and construct tissue-bound conductive polymers through the oxidative polymerization of precursors, offering valuable insights for the development of multimodal soft electrodes within living organisms. Additionally, after modifying HA with tyramine, an injectable hydrogel can be constructed through a cascade catalysis involving HRP and GalOX, which avoids the biotoxicity caused by excessive H_2_O_2_ introduced from the external environment and the heterogeneity resulting from rapid gelation.^132^ Furthermore, functional DNAzyme hydrogels were developed by integrating peroxidase-mimicking DNAzyme into DNA motifs. Encapsulating GOx and β-galactosidase within the DNAzyme hydrogel enabled the detection of glucose/lactose through hybridization cascade enzymatic reactions.^133^

**Figure 8:**
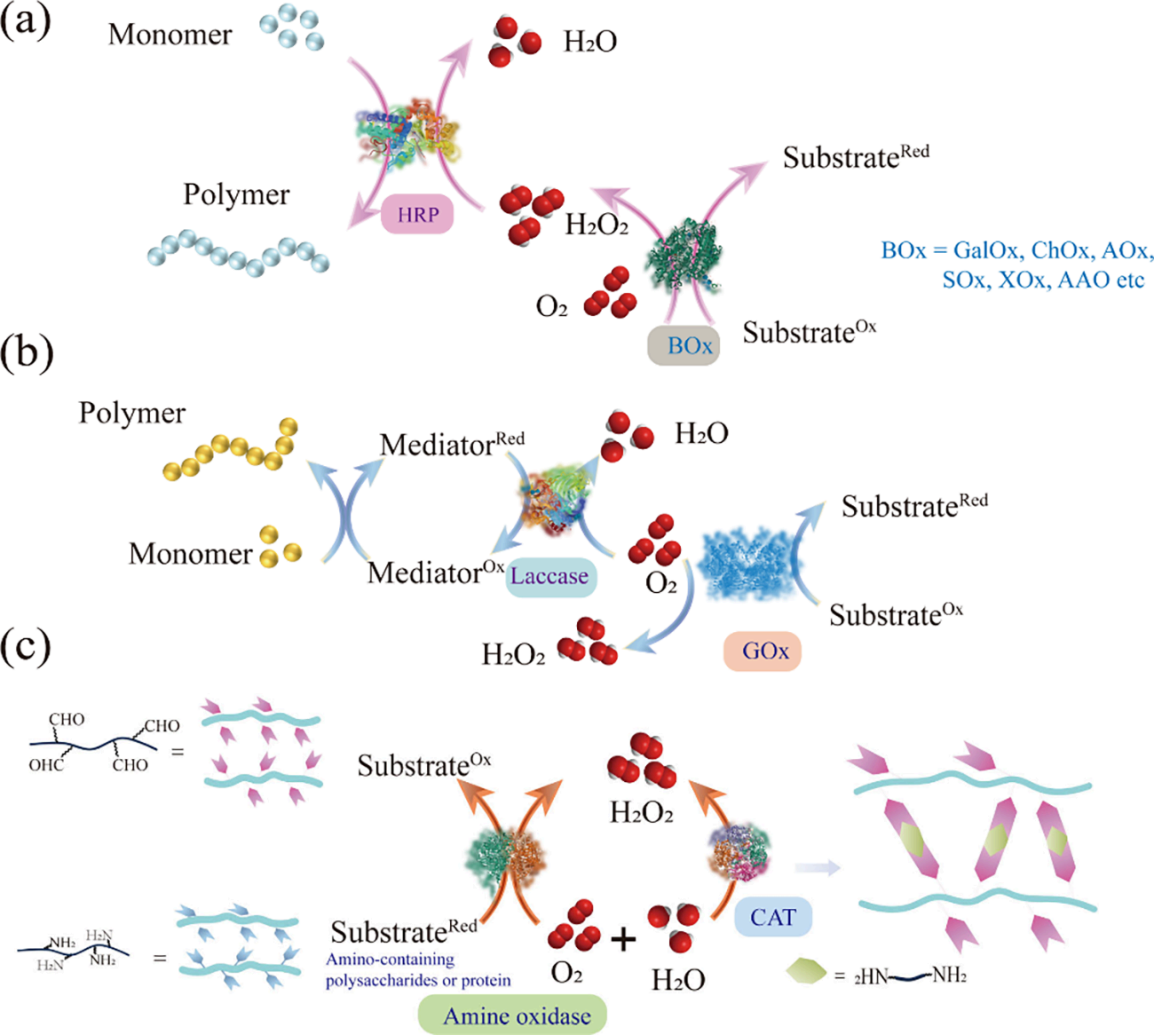
(a) Schematic illustrating the oxidase/HRP cascade catalysis system for maintaining H_2_O_2_ concentration balance and initiating monomer polymerization. (b) Schematic of the GOx/laccase cascade catalysis system for oxygen concentration regulation and monomer polymerization mediation. (c) Catalytic process after deamination of amino-functionalized polysaccharides/proteins by monoamine oxidase (MAO) and subsequent polymerization with long-chain amino compounds. Peroxidase mediates H_2_O_2_ decomposition to regulate oxygen levels while avoiding hydrogen peroxide byproducts.

**Figure 9:**
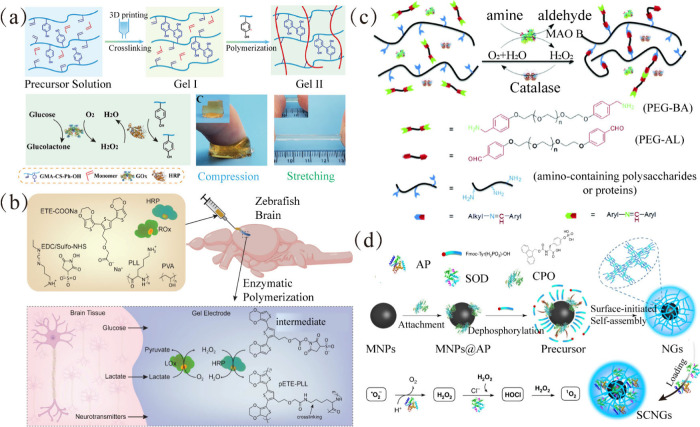
(a) Composite hydrogels with enhanced mechanical properties were fabricated though a strategy of prepolymerization followed by cross-linking mediated by GOx/HRP cascade catalysis. Adapted with permission from ref 130. CC BY 4.0. (b) pETE-PLL conductive polymer hydrogel electrodes were prepared via the induction of endogenous metabolites, which lead to the polymerization and cross-linking of ETE monomers mediated by the LOx/HRP cascade system. Reproduced with permission from ref 131. Copyright 2023 the authors of ref 131, exclusive licensee American Association for the Advancement of Science. (c) The MAO and CAT cascade system catalyzes oxidative deamination reactions to form PEG-AL, which when reacted with GC or gelatin through the Schiff base reaction prepares dynamically responsive hydrogels with excellent biocompatibility. Reproduced with permission from ref 136. Copyright 2017 Royal Society of Chemistry. (d) Enzymatic dephosphorylation by ALP initiates the self-assembly of oligopeptides around nanoparticles, leading to the formation of nanogels that immobilize the SOD and CPO cascade enzyme system. Reproduced from ref 137. CC BY 4.0.

A cascade enzyme system not only regulates the concentration of H_2_O_2_ but also addresses the issue of oxygen balance within the reaction system, thereby creating an optimal environment for catalysis polymerization. Specifically, the GOx and laccase cascading system plays a crucial role in the preparation of enzyme-laden hydrogels. This system ensures the dual functionality of oxygen, acting as an oxidizing agent in the laccase-mediated reaction and preserving radicals and chain growth during the polymerization process (Figure 8b). For instance, a PEG-*g*-F68 hydrogel was obtained by employing a copolymer of poly­(ethylene oxide)–poly­(propylene oxide)–poly­(ethylene oxide) block copolymers as the mediator and the diacrylic derivative of poly­(ethylene glycol) as the monomer in a GOx/laccase cascade system to consume excessive oxygen during glucose oxidation, thereby maintaining appropriate oxygen levels and achieving reasonable polymer conversions in the enzymatic polymerization process.^134^

Parallel cascade reactions are a prevalent mechanism in biocatalytic redox processes. This concept is practically applied in the preparation of enzyme-laden hydrogels, wherein the product formation is combined with a simultaneous secondary reaction. A typical example is the cofactor cycling of NAD­(P)­H, which relies on redox enzymes.^135^ One notable application of parallel cascades in hydrogel preparation involves systems where a primary reaction produces a toxic byproduct, which is then decomposed by a secondary enzymatic reaction. A key example is the use of catalase (CAT) to convert toxic hydrogen peroxide byproducts into water and oxygen, significantly enhancing the biomedical applicability of the hydrogels (Figure 8c). For example, Wei et al. utilized the oxidative deamination catalyzed by monoamine oxidase B to convert amino groups on benzylamine-functionalized poly­(ethylene glycol) (PEG-BA) into aldehyde groups (PEG-AL), followed by reaction with ethylene glycol chitosan (GC) or gelatin (collagen) via Schiff base reaction to construct self-healing hydrogels. The byproduct hydrogen peroxide is then decomposed through a CAT-mediated reaction (Figure 9c).^136^

A bienzymatic cross-linking approach was employed to prepare interpenetrating biopolymer hydrogel fibers through wet spinning. One network consisted of gelatin catalyzed by microbial transglutaminase (mTG), while the other network was formed by chitosan grafted with phloretic acid (chitosan-PA), induced by horseradish peroxidase (HRP) in the presence of H_2_O_2_.^135^ Additionally, a novel cascade enzyme microgel was synthesized by Wu et al. that attached alkaline phosphatase ALP to the core of metal nanoparticles, where ALP catalyzed the dephosphorylation of hydrophilic peptide precursors, leading to peptide self-assembly and the immobilization of SOD and CPO (Figure 9d). This surface phosphatase-triggered self-assembly of oligopeptides around nanoparticles induces the production of neutrophil lysosomes, while the SOD and CPO enzyme cascade upregulates ROS, thus targeting tumor treatment.^137^

Cascade-enzyme-catalyzed reactions offer several advantages, including more compatible reaction conditions, reduced instability of the intermediates, and fewer side reactions. These benefits make enzymatic cascade catalysis particularly attractive in industries such as pharmaceuticals and the synthesis of novel compounds. However, the application of cascade enzymes in developing enzyme-laden hydrogels is still limited, with only a few enzyme species and types currently in use. For optimal enzymatic activity, cascade-enzyme-catalyzed reactions must occur in a mild and favorable environment. Therefore, expanding the range of cascade enzymes and engineering enzymes with enhanced environmental tolerance are crucial for advancing the construction of enzyme-laden hydrogels.

#### 2.2.3 Mimic-Enzyme Catalysis Systems

Inherent limitations, including high purification costs, low yields, and poor stability under specific environmental conditions, impose significant constraints on the applications of natural-enzyme-based hydrogel constructs. Conversely, since the term “nanozymes” was coined following the pioneering work of Yan, who first reported that Fe_3_O_4_ nanoparticles exhibited peroxidase-like activity,^138^ research on nanozymes has surged. This active period of study is propelled by the advantageous attributes of mimic enzymes, including their simple structure, high durability, low cost, strong environmental tolerance, and stable chemical properties.^139–141^ Currently, the composition of mimic enzymes can be categorized into several types: metal-based nanozymes that mimic the activity centers of natural enzymes, such as noble metal nanozymes like gold and palladium, metal oxide nanozymes like MnO_2_ and CeO_2_, and amino acid-coordinated metals such as glycine iron and lysine iron; metal–organic frameworks; and non-metal-based nanozymes, primarily composed of carbon-based materials and natural protein compositions.^142–144^ Moreover, these mimic enzymes exhibit activities akin to those of the oxidase family (e.g., catecholase, laccase), the peroxidase family (e.g., horseradish, NADH peroxidases), superoxide dismutase, CAT, phosphatase, and multienzyme systems (Figure 10).^145–148^

**Figure 10:**
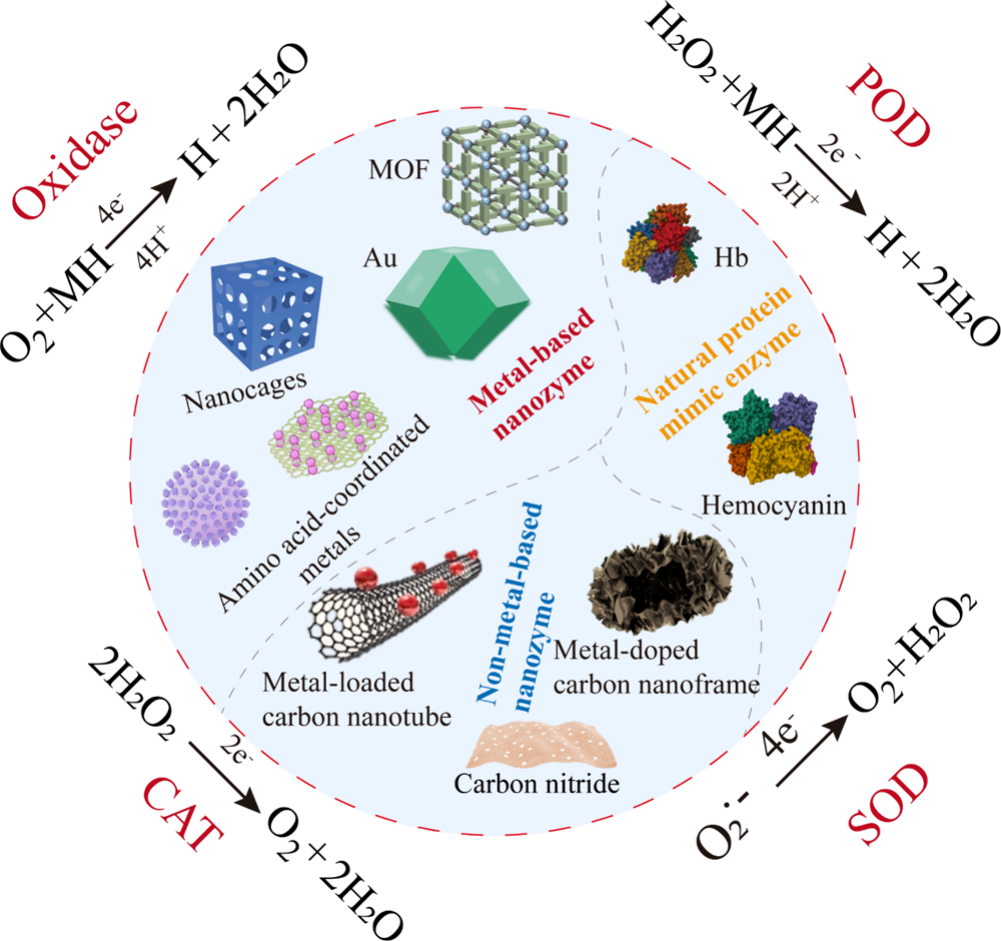
Materials commonly used for mimicking enzymes and their corresponding enzyme-like activities.

After the discovery that iron magnetic nanoparticles possess peroxidase-like activity, metal materials have become a focal point in enzyme mimicry research. For instance, based on the HRP-like activity of CuO nanoparticles, researchers designed a GOx/CuO cascade system in which the H_2_O_2_ generated by GOx undergoes a Fenton-like reaction with CuO, producing hydroxyl radicals and initiating monomer polymerization to prepare a transparent nanozyme gel (Figure 11a).^149^ Furthermore, Qu’s group has conducted more research on simulating enzymes, reporting the preparation of MoS_2_-hydrogel scaffolds with peroxidase catalytic properties by covalent cross-linking of MoS_2_ nanoflowers with hydrogels. These modified nanogel materials, with excellent biological safety, utilize the features of MoS_2_ in the NIR region, including intrinsic peroxidase activity and high photothermal conversion efficiency, providing a superior option for effective bacteria elimination.^29^ Additionally, a multienzyme catalytic system constructed from natural enzymes and simulated enzymes can enhance catalytic efficiency and modulate the structure and mechanical properties of hydrogels. Using acryloyl-modified chondroitin sulfate (CS) and *N*,*N*-dimethylacrylamide (DMAA) as monomers, in the composite enzyme system Fe­[Gly]_2_, with HRP-like activity, internalizes the glycine ligand, generating carbon radicals through decarboxylation. This process triggers rapid and efficient monomer polymerization (within 5 s) for gelation (Figure 11b).^150^ The creation of an optimized diffusion environment for cascade enzymes and external small-molecule channels by initiating polymerization at the interface of nanomaterials has also been reported. Using burr-defect-engineered molybdenum disulfide/Prussian blue analogues (Burr-NCs) as the core, monomer polymerization is triggered based on the peroxidase and Fenton-like catalytic activity of the core, forming a hydrogel shell network. Superoxide dismutase (SOD) is then encapsulated in the shell layer to achieve efficient cascade catalytic reactions for in vivo therapeutic applications (Figure 11c).^151^ The confined hydrogelation strategy on the nanointerface offers valuable insights into the biological assembly of simulated enzymes while presenting a flexible approach for enhancing the functional properties of nanomaterials. MOFs, with their porous and diverse structures, are suitable for enzyme immobilization, and their transition metals can serve as biomimetic catalysts, making MOF functional nanomaterials significant in enzyme mimic construction. For example, the construction of a peroxidase mimic as an ATRP catalyst by grafting heme onto the MOF UiO-66-NH_2_ (ZrMOF) with 1,1-carbonyldiimidazole (CDI) as an activator has been reported.^152^ Anther study mentioned the enzyme mimic deuterohemin-β-Ala-His-Thr-Val-Glu-Lys (DhHP-6), consisting of six amino acid residues and one iron porphyrin with high peroxidase activity, which was embedded in ZIF-8 via a biomimetic mineralization method and utilized for ATRP polymer synthesis.^153^ Natural enzymes, primarily composed of proteins with a small number of RNA molecules, are highly effective biocatalysts. Reports indicate that certain proteins such as hemoglobin, hemocyanin, and myoglobin can serve as enzyme mimics for biocatalysis. It is not uncommon to find articles utilizing hemoglobin as a peroxidase mimic to cross-link tyrosine residues in silk fibroin.^98,154,155^ Additionally, one study reported the use of HbO_2_ as a catalyst for forming horseradish peroxidase-mediated hydrogels and as an oxygen carrier to alleviate hypoxia. Under near-infrared-irradiation-induced mild heating, oxygen can be released in a controlled manner. Artificial non-enzyme antioxidant MXene nanosheets can eliminate excessive reactive nitrogen and oxygen species, maintaining cellular redox homeostasis and reducing oxidative stress (Figure 11d).^156^

**Figure 11:**
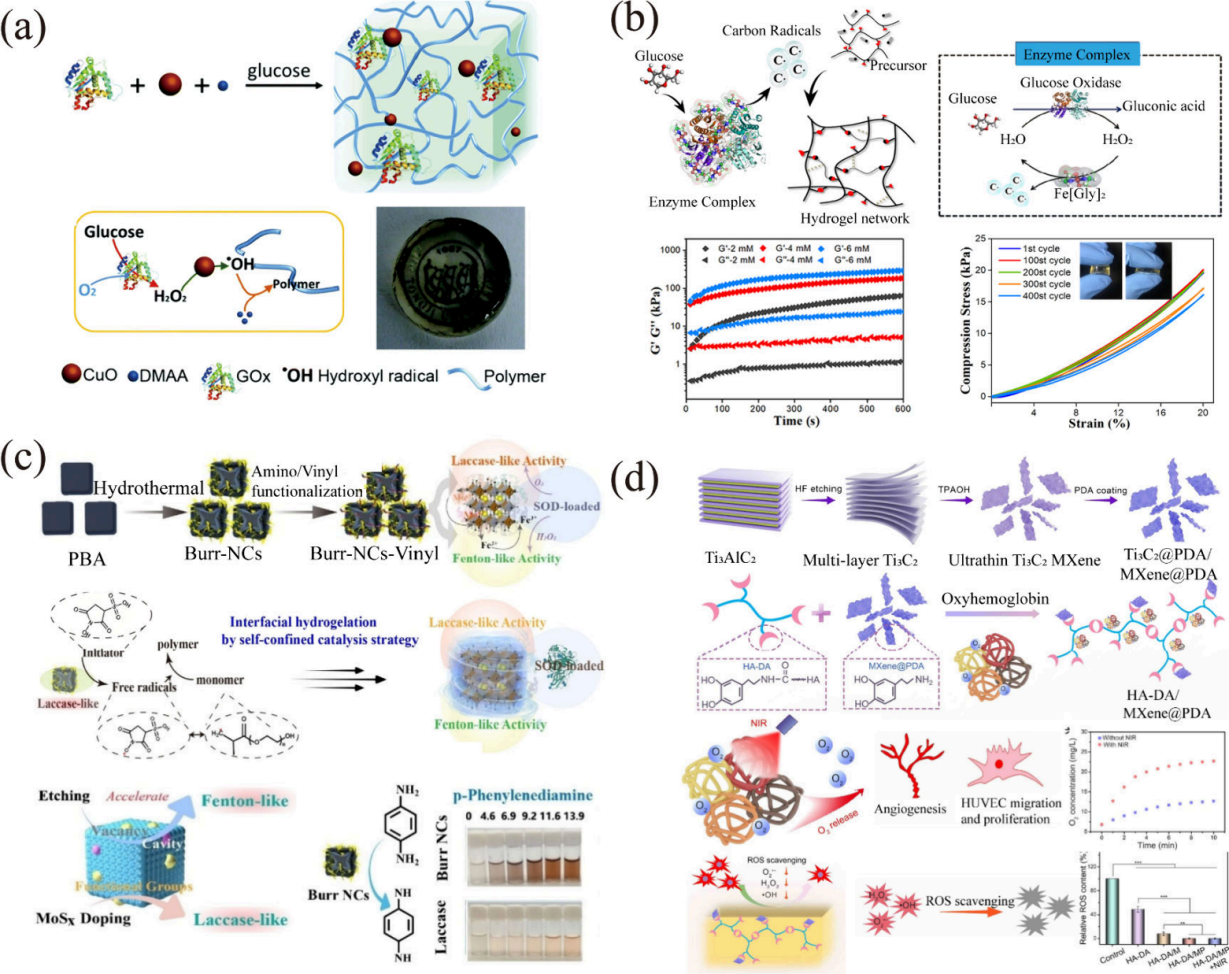
Construction of enzyme-integrated hydrogels using enzyme mimics. (a) CuO NPs were selected as mimics for HRP to facilitate free radical polymerization and gelation in the presence of GOx. Reproduced with permission from ref 149. Copyright 2017 Royal Society of Chemistry. (b) The HRP-mimetic properties of Fe­[Gly]_2_ involve internalization of the glycine ligand and subsequent access to carbon radicals through decarboxylation, resulting in polymerization and hydrogelation. Reproduced with permission from ref 150. Copyright 2021 Wiley-VCH. (c) SOD-incorporating microgels constructed through interface polymerization of Burr-NCs, which exhibit peroxidase-like activity and Fenton-like catalytic behavior. Reproduced with permission from ref 151. Copyright 2023 Wiley-VCH. (d) HbO_2_ acts not only as an HRP-like enzyme to catalyze the oxidative coupling of catechol groups for hydrogel fabrication but also as an oxygen carrier to control oxygen release induced by heat generated from NIR irradiation. Reproduced from ref 156. Copyright 2022 American Chemical Society.

Mimic enzymes exhibit numerous advantages, including stability, facile synthesis, and tolerance toward environmental conditions, which address the drawbacks of natural enzymes, which are easy to deactivate and challenging to prepare.^157,158^ Currently, in addition to nanomaterial-based mimics, traditional mimics such as cyclodextrin mimics,^159,160^ porphyrin mimics,^161,162^ molecularly imprinted polymer mimics,^163,164^ and micelle mimics,^165^ have undergone extensive investigation across various disciplines, offering novel insights for the prospective development of gel-integrated enzymes.

#### 2.2.4 Physical–Biochemical Coupling Catalysis for Constructing Enzyme-Integrated Hydrogels

Photosynthesis, the most extensive solar energy utilization system on Earth, provides a blueprint for developing light–biomolecule coupling systems that convert solar energy into chemical energy. Light also serves as a highly convenient external stimulus, enabling precise spatiotemporal control over systems regulation.^166^ Biological enzymes offer exceptional catalytic efficiency, environmental friendliness, and strong specificity. However, most enzymatic reactions are thermally activated and constrained by rates of substrate/coenzyme binding, product release, and protein conformational changes.^167,168^ The growing demand for green, efficient, and sustainable synthesis has spurred innovation in photoenzymatic catalysis, which integrates light with biocatalysts.^169–172^ While naturally occurring light-activated enzymes (e.g., protochlorophyllide oxidoreductase, fatty acid photodecarboxylase, and DNA photolyase) are limited,^173–175^ studies demonstrate that flavoproteins (utilizing flavin adenine dinucleotide (FAD) or flavin mononucleotide (FMN) cofactors derived from vitamin B_2_)^176^ can be coupled with light for green, sustainable, and controllable biocatalysis. For instance, An’s group developed a non-natural photoenzymatic process using flavoprotein GOx under visible light to drive reversible addition–fragmentation chain transfer (RAFT) polymerization.^177,178^ Similarly, Hyster and colleagues employed light-activated flavin ene reductase for stereoselective radical cyclization, providing a foundation for novel enzyme-catalyzed organic syntheses.^179^

Despite its promise, photoenzymatic catalysis faces challenges including enzyme stability, light-induced decomposition of critical substrates (e.g., NADH), and the generation of free radical intermediates/byproducts that can lead to enzyme inactivation.^180^ To overcome these limitations, the construction of hydrogels via a physical–biochemical coupling system offers an effective strategy. Among these, photo–enzyme coupling systems represent a prominent approach. As illustrated in Figure 12a, controllable light activates enzymes to generate radicals, which subsequently initiate monomer polymerization and cross-linking preferentially around the enzyme. This localized reaction forms a network that encapsulates the enzymes, effectively preserving their catalytic function. Building on this principle and inspired by photosynthesis, Wang’s group developed a mild, efficient, and tunable physical–biochemical (light–enzyme) coupling catalytic system. This system employs a coupling mechanism where light-induced electron transfer occurs with the enzyme’s active site via proton-coupled electron transfer, enabling potential light-activated redox regulation. (Figure 13a)^181^ Furthermore, radicals generated by photoactivated CAT were harnessed to initiate monomer polymerization, constructing enzyme-laden bioactive hydrogels for spatiotemporally controlled enzyme immobilization (Figure 13b).^182^ Crucially, the radical polymerization occurs locally around the enzyme, effectively immobilizing it while preserving high catalytic activity. Notably, UV–vis spectrophotometric analysis confirmed that the encapsulated enzymes exhibited significantly enhanced stability under harsh acidic (pH 4) or alkaline conditions (pH 9) compared to their free counterparts—as evidenced by greater absorbance changes indicating higher activity in the enzyme-integrated hydrogel—thereby expanding the operational versatility of this photo–enzyme coupled system within challenging microenvironments.

**Figure 12:**
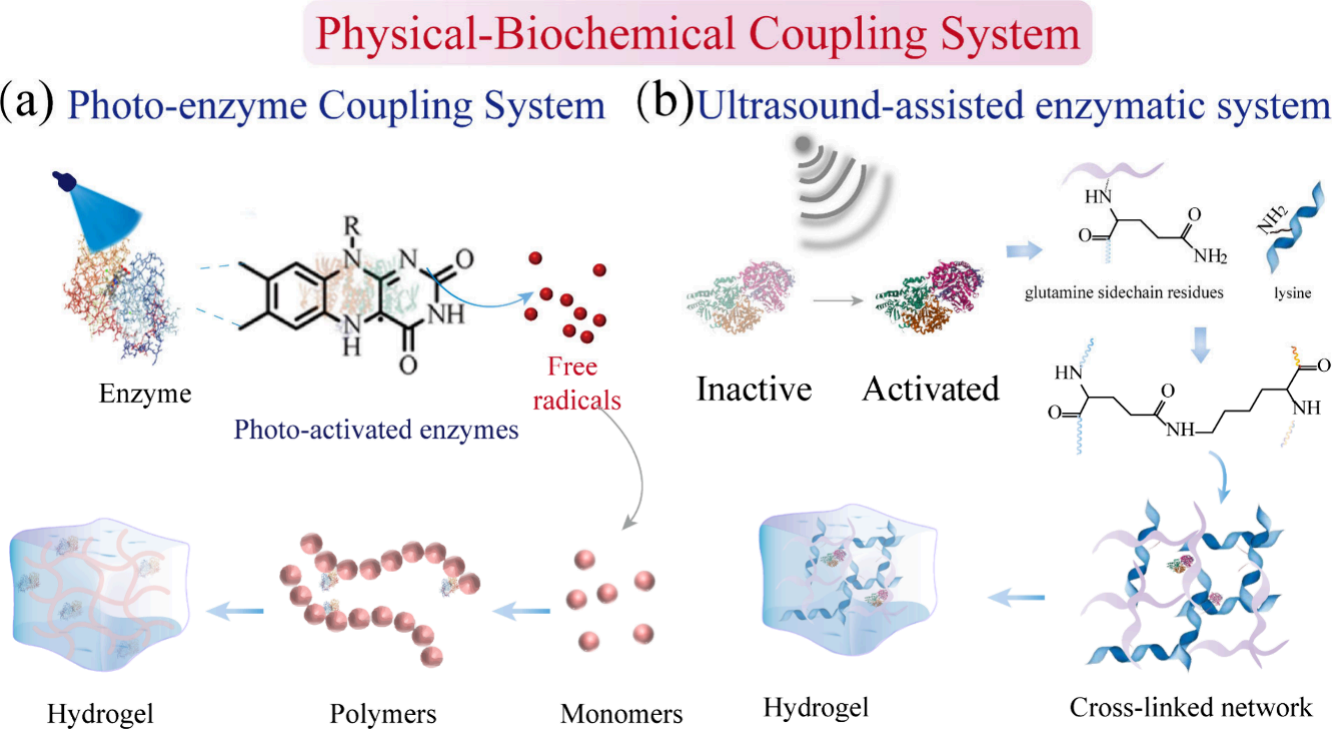
Physical–biochemical coupling strategy for the fabrication of enzyme-functionalized hydrogels. (a) Photo–enzyme coupling system and (b) ultrasound-assisted enzymatic system for constructing enzyme-integrated hydrogels.

**Figure 13:**
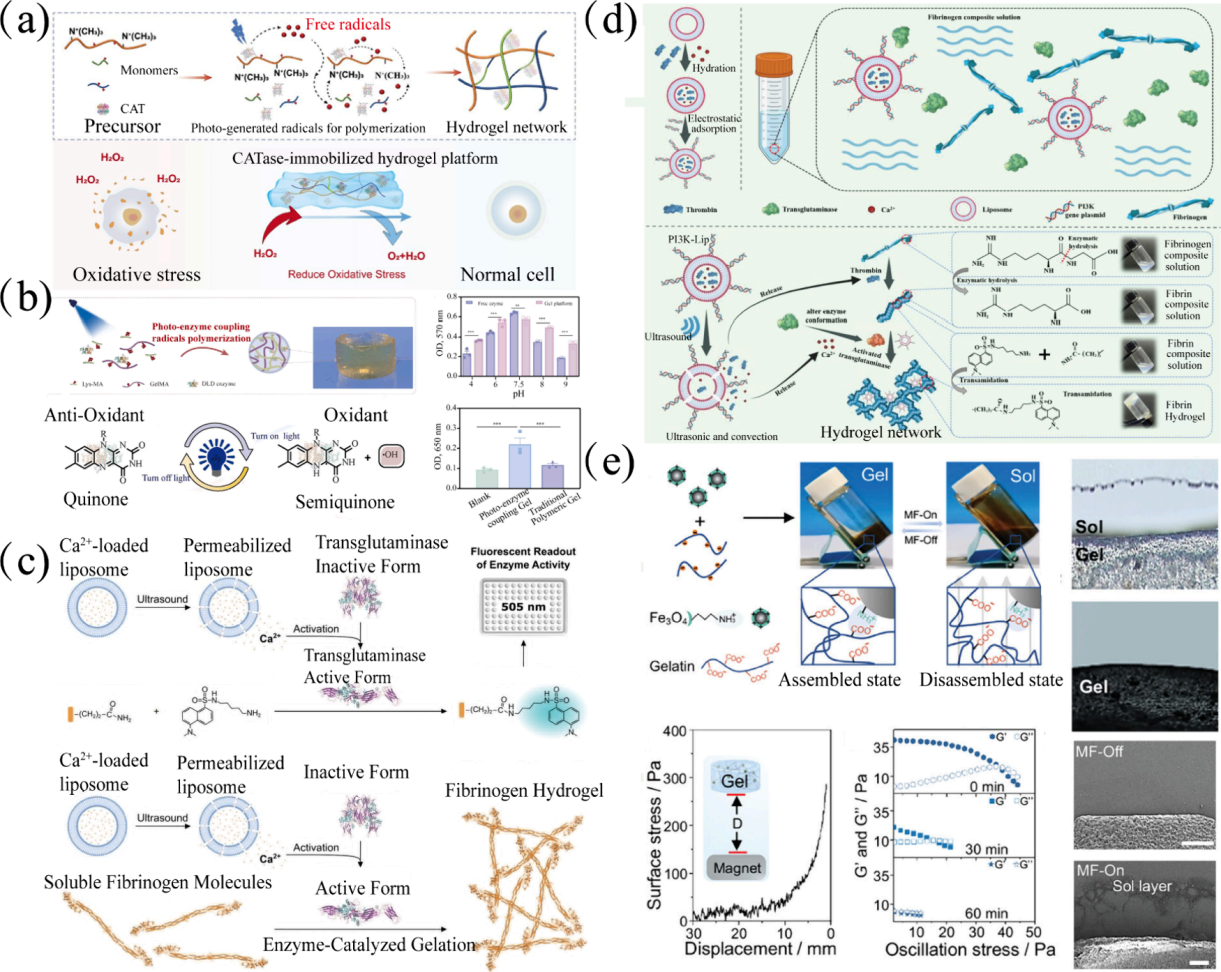
Construction of enzyme-integrated hydrogels via physical–biochemical coupling systems. (a) Blue-light-activated flavoenzyme generates free radicals to initiate monomer polymerization, resulting in the fabrication of an enzyme-integrated hydrogel. Reproduced with permission from ref 181. Copyright 2023 Elsevier. (b) UV-activated catalase generates radicals to form a three-dimensional network for enzyme immobilization. Reproduced with permission from ref 182. Copyright 2023 Science China Press. (c) Schematic of ultrasound-triggered transglutaminase-mediated hydrogelation. Reproduced from ref 183. CC BY 4.0. (d) Tissue-penetrating ultrasound-triggered hydrogel network fabricated using calcium ions and transglutaminase as catalysts. Reproduced from ref 184. CC BY 4.0. (e) Magnetic field switching induces the Fe_3_O_4_–NH_2_ nanoparticles to decompose the triple helix structure of gelatin, thereby effecting the sol–gel transition in the hydrogel. Reproduced with permission from ref 187. Copyright 2024 Wiley-VCH.

Beyond light, other physical stimuli, such as ultrasound and magnetic fields, also offer power strategies for constructing enzyme-integrated hydrogels, albeit often through distinct mechanisms. In ultrasound-triggered hydrogelation systems, inactive enzymes are activated by ultrasound, catalyzing intermolecular covalent cross-linking between the lysine and glutamine residues of monomers (Figure 12b). Critically, the physicochemical properties of the resulting enzyme-integrated hydrogel can be precisely modulated by the physical exposure parameters. For instance, the Stevens group demonstrated that ultrasound could release calcium ion from liposomes, subsequently activating TGase to catalyze the covalent cross-linking of fibrinogen into hydrogels (Figure 13c).^183^ By modulating the ultrasound duration, they achieved control over enzyme kinetics and resultant hydrogel properties, establishing ultrasound as a viable remote trigger for in vivo gelation. Similarly, Zuo et al. reported a tissue-penetrating ultrasound-triggered hydrogel formation strategy (Figure 13d).^184^ This approach employs ultrasound to induce liposome-mediated thrombin release into solution. Thrombin then hydrolyzes fibrinogen, facilitated by calcium ions and TGase as catalysts, to form in situ a fibrin-based solid hydrogel network that promotes microvascular network regeneration within tissues.

Magnetic fields, while less commonly used to directly initiate enzyme-mediated polymerization compared with light or ultrasound, are frequently leveraged in conjunction with physical or chemical cues to achieve sophisticated therapeutic outcomes. Unlike photoactivated enzymes that generate localized free radicals, magnetic fields often work synergistically. For example, Mano et al. encapsulated collagenase with magnetic nanoparticles via an oil-in-water emulsion method to fabricate microgels enzymatically. These microgels, under external magnetic guidance, enable precise sculpting of microchannel structures within biomimetic hydrogels.^76^ Zhang et al. developed magnetic hydrogel nanozymes (MHz) by exploiting host–guest interactions between poly­(ethylene glycol)-functionalized nanoparticles and α-cyclodextrin, forming supramolecular hydrogels with shear-thinning and temperature-sensitive phase transition properties. Upon exposure to alternating magnetic fields, these MHz enhance tumor therapy efficacy through the synergistic actions of magnetically induced hyperthermia and nanozyme catalysis.^185^ Huang et al. further integrated poly­(ethylene glycol)/polyethylenimine-modified superparamagnetic nanoparticles (SPIONs) and aptamer-modified palladium hydride nanoenzymes (PdH-Apt) into polyacrylamide/hyaluronic acid (PAAm/HA) hydrogels, creating a superparamagnetically responsive system.^186^ This hydrogel leverages combined physical and chemical cues to temporally regulate endogenous stem cell fate, thereby promoting intervertebral disc regeneration. Moreover, magnetically responsive semiconvertible hydrogels capable of undergoing sol–gel transition upon magnetic field switching were designed by Gao et al.^187^ This system exploits the weak non-covalent bonds formed via electrostatic interactions between Fe_3_O_4_–NH_2_ nanoparticles and negatively charged gelatin chains. Upon application of a magnetic field (MF), these Fe_3_O_4_–NH_2_ nanoparticles induce decomposition of the gelatin triple-helix structure, resulting in partial gel–sol transition within the hydrogel (Figure 13e).^187^ This design strategy holds promise for developing actuators based on intelligent hydrogel systems that incorporate magnetically active soft materials.

Collectively, physical stimuli (e.g., light, sound, and magnetic fields) offer pathways to construct enzyme-integrated hydrogels, aligning with green chemistry principles. This innovative approach enhances biocompatibility and enables precise structural design with spatiotemporal control, making it highly suitable for personalized tissue therapies. However, several challenges remain, including potential thermal and mechanical damage induced by ultrasound that may compromise structural precision as well as the need for further investigation into the safety of light sources and photoreactions for in vivo applications. Further advancements in the functionality of enzyme-integrated hydrogels will likely rely on the integration of multiple technologies, the development of electricity-activated enzyme polymerization techniques, and the strategic combination of physical stimuli with enzymatic advantages.

#### 2.2.5 Other Methods for Constructing Enzyme-Integrated Hydrogels

In addition to the aforementioned methods, covalent bonding immobilization, intelligently responsive hydrogel immobilization techniques, genetic fusion proteins, and hydrogel-based 3D-printed enzyme reactors have attracted attention for their simplicity and efficiency in constructing enzyme-integrated hydrogels.^16,188,189^ Among these techniques, covalent bonding immobilization, which utilizes the reactive groups of enzyme molecules—such as amino, carboxyl, or thiol groups—to form stable covalent bonds with functional groups present in the hydrogel, ensures strong enzyme–hydrogel binding, thereby providing high immobilization stability and minimizing enzyme leakage. For example, Zheng et al. covalently incorporated thrombin and hirudin enzyme into a hydrogel network, enabling the reversible activation and deactivation of biocatalytic activity by applying or removing mechanical stretching, which disrupted or restored the nanocovalent interactions between thrombin and hirudin, offering a biomacromolecular strategy for mechanoregulated soft functional materials.^78^ Intelligent responsive hydrogels possess the capability to modify their structure or properties in response to external environmental stimuli (e.g., temperature, pH, or light), and when enzymes are immobilized within these hydrogels, their activity can be regulated by such stimuli, enabling the “on-demand release” or “on-demand activation” of enzymatic functions. Shang et al. synthesized nanozymes (AuNCs) using l-3,4-dihydroxyphenylalanine as the functional ligand, which upon reacting with phenylboronic acid-modified sodium alginate to form boronate ester bonds enabled a sensitive response to ROS and glucose, allowing for controlled degradation and responsive release of the Au nanozymes, thereby achieving both antioxidant effects and immune modulation.^190^

The fabrication of enzyme-integrated hydrogels employs diverse methodologies. Conventional strategies, such as physical entrapment and adsorption, offer significant advantages in terms of simplicity and cost-effectiveness. Crucially, these methods typically preserve the enzyme’s native conformation by minimizing exposure to harsh chemical conditions, thereby acting as a protective barrier. This barrier enhances enzymatic stability during operation while concurrently reducing the risks of denaturation and leaching. However, achieving sustained high catalytic activity often necessitates covalent immobilization or sophisticated encapsulation techniques. Theses chemical and advanced physical approaches impose stringent requirements on the reaction environment, demanding precise control over pH, temperature, ionic strength, and the use of biocompatible cross-linkers to maintain enzyme integrity and function. Similarly, purely physical methods face inherent limitations, including inadequate mass transfer rates that create substrate diffusion barriers and lead to reaction inefficiencies coupled with intrinsically low and unstable enzyme loading capacities. Looking ahead, pivotal research efforts must focus on elucidating the complex interplay between key fabrication parameters—such as cross-linking density, network architecture, and microenvironmental cues—and the resulting hydrogel’s biophysicochemical properties. Concurrently, innovation in noninvasive, spatiotemporally controlled integration techniques—exemplified by bio-orthogonal chemistry, advanced stimuli-responsiveness, or four-dimensional bioprinting—is essential. The ultimate goal remains the development of robust, scalable platforms that ensure both high enzymatic efficiency and long-term operational stability for demanding biomedical and biocatalytic applications.

## 3 Applications of Enzyme-Integrated Hydrogels

Benefiting from their extracellular matrix-like structure, high water content, and tunable mechanical properties, hydrogels hold significant promise for diverse applications, including tissue engineering, cell culture, regenerative medicine, drug delivery, soft robotics, biosensing, and bioelectronics. Specifically, enzyme-integrated hydrogels enable novel biodiagnostic systems and therapeutic strategies. These integrated systems facilitate rapid and accurate target detection, exemplified by glucose sensing, through enzyme-mediated electron–ion transfer channels within reconstituted aerobic metabolic processes. Concurrently, the enzymatic activity embedded within these hydrogels provides a biotherapeutic strategy, intervening in the aerobic metabolic pathways of diseased cells and thereby offering a treatment strategy modality targeting the maintenance of redox homeostasis.

### 3.1 Enzyme-Integrated Hydrogels in Medical Diagnostics

Enzyme diagnosis, a widely used method employing enzymatic catalysis to analyze specific substances within the body, plays a crucial role in disease diagnosis. Techniques such as enzyme-linked immunosorbent assay (ELISA) and sol–gel-based enzyme biosensors are prevalent in clinical enzyme diagnostics, enabling the detection of high concentrations of ions, biomolecules, and relevant biomarkers in various biological fluids, including sweat, tears, urine, serum, and plasma, as well as organ tissues.^191,192^ Notably, wearable and implantable enzyme-integrated hydrogel sensors have garnered significant attention for detection of small biomolecules such as glucose, lactate, urea, cholesterol, and pathogens as well as for use in wound bioassays and are favored for their biocompatibility, adhesiveness, ease of processing, tissue-like mechanical properties, rapid response, and capacity for real-time extraction of biomarkers from tissues.^193–195^ Specific examples demonstrate these advantages. A dopamine biosensing platform was developed by encapsulating TYR within a CdTe quantum dot (QD) hydrogel network via sol–gel technology, achieving a detection limit for dopamine 4 times lower than that of TRS-CdTe QDs.^193^ A new urease-functionalized inverse opal hydrogel (IOHP) sensor was created for detecting and eliminating urea, achieving detection up to 30 mM with a limit of 0.48 mM, facilitated by the hydrogel’s carboxyl groups for urease immobilization and pH-responsiveness.^196^ Wu et al. developed an implantable hydrogel platform incorporating luminescent polymer dots (Pdots) for long-term glucose monitoring,^197^ utilizing a biocoupled system of GOx and oxygen-sensitive Pdots. Here GOx consumes oxygen during glucose oxidation, generating a light signal correlated with the glucose concentration, and the sensitivity is tunable by varying the enzyme concentration and injection amount (Figure 14a).^197^ Additionally, a borate fluorescence intensity hydrogel glucose sensor coloaded with SOD and CAT enzymes demonstrated clinically relevant accuracy for tracking blood glucose concentrations in rats for up to 5 h.^198^

**Figure 14:**
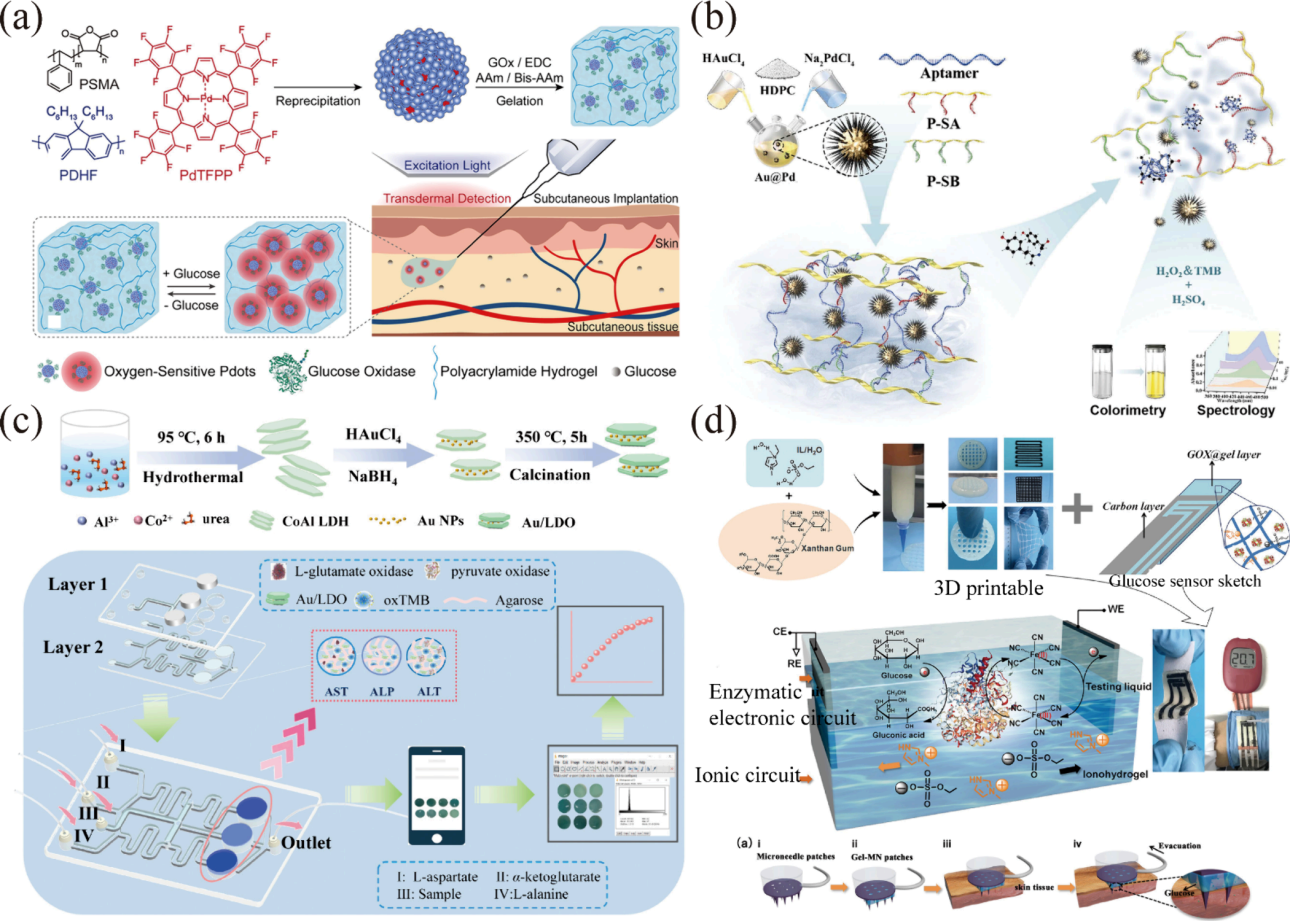
Enzyme-integrated hydrogel sensors for monitoring biochemical indicators. (a) The reprecipitation synthesis of Pdots (using a semiconducting polymer and phosphorescent dye) produced a hydrogel–Pdot transducer for successful transdermal optical detection of glucose in vivo. Reproduced from ref 197. Copyright 2022 American Chemical Society. (b) Schematic of a colorimetric biosensor for ractopamine (RAC) detection based on a DNA hydrogel encapsulating Au@Pd nanoparticles. Reproduced with permission from ref 200. Copyright 2023 Elsevier. (c) Fabrication of Au-decorated CoAl-layered double oxide (Au/LDO) nanozyme and the hydrogel-involved colorimetric platform for point-of-care testing of liver-related biomarkers. Reproduced from ref 201. Copyright 2022 American Chemical Society. (d) Integration of an enzymatic electronic circuit that relies on the biocatalytic oxidation of glucose and an ionic circuit based on hydrated ionic liquids into an ionohydrogel platform for glucose detection. Reproduced with permission from ref 202. Copyright 2020 Wiley-VCH.

Beyond natural enzymes, significant research focuses on integrating artificial enzymes into hydrogel frameworks to establish chromogenic platforms for detecting enzymes and biomarkers. Lu and colleagues fabricated stable, mussel-inspired nanozymes (TA-Ag) by chelating ultrasmall Ag nanoparticles with tannic acid^199^ and leveraged their peroxidase-like (POD) activity to trigger hydrogel self-setting, endowing the hydrogel with antibacterial, conductive, and adhesive properties for bioelectronics. A stable colorimetric sensing system platform for sensitive detection of ractopamine (RAC) was established using a DNA hydrogel encapsulating Au@Pd nanoparticles.^200^ Cross-linking via responsive DNA causes the hydrogel to collapse upon RAC addition, releasing Au@Pd NPs, which catalyze a colorimetric reaction (Figure 14b). This yields a detection limit of 7.39 ng L^–1^ and a range of 0.01 to 1000 μg L^–1^, demonstrating significant potential for practical RAC detection.^200^ Furthermore, an innovative colorimetric sensor was fabricated by integrating hybrid hydrogels into a miniaturized device for detecting liver-related indicators such as AST, ALT, and ALP.^201^ Au-decorated CoAl-layered double hydroxide (Au/LDO) nanoparticles with POD activity and reactants were immobilized in the hydrogel, and the sensor relies on smartphone-based visual signal output (Figure 14c). This disposable hydrogel design allows substitution for detecting various chemical/biological targets.

Accurate and rapid diagnosis is paramount to effective disease treatment. However, current enzyme diagnostic techniques often involve time-consuming sampling, overlook the need for reliability and speed, and lack high enzyme activity retention and immediate in situ detection capabilities. To address these challenges, Wang and colleagues developed a gel-based bioplatform leveraging electronic transfer and signals within simulated aerobic metabolism, which addresses issues of enzyme activity maintenance and ion migration, enabling rapid in situ glucose detection (Figure 14d).^202^ Following the Hofmeister series, 1-ethyl-3-methylimidazolium ethylsulfate ([EMIM]­[EtSO_4_]) was selected as a multifunctional solvent for enzymatic polymerization, facilitating gelation and in situ enzyme immobilization to synthesize an ionohydrogel bioelectronic platform. This integration merges ionic and enzyme electronic circuits, addressing tissue–device mismatch, ensuring high immobilized enzyme bioactivity, and serving as an ion conductor for human motion monitors and an enzyme electronic conductor for glucose biosensors.

Enzyme-integrated hydrogels possess compressible hydrophilic networks, excellent biocompatibility, designability, and adjustable mechanical properties, rendering them advantageous not only as biosensors for prompt biosignal detection but also for various imaging applications such as ultrasonic imaging, fluorescence imaging, photoacoustic imaging, and magnetic resonance imaging due to enhanced backscattering signals and improved visualization. For instance, Wang et al.^203^ developed a mesoporous silica nanoparticle (MSN)-based nanogel via in situ amidation-fueled self-assembly of peptide gelators (NapFFK, NapFFK-acrylic) and enzymatic polymerization, creating a multifunctional nanotheranostic agent for ultrasound imaging and imaging-guided high-intensity focused ultrasound (HIFU) therapy whose fluorescence is activated by the tumor microenvironment’s low pH and high H_2_O_2_ levels (Figure 15a). Qi et al. prepared Fe ion-ligated aryllysine polymer brushes via enzyme-catalyzed atom-transfer radical polymerization, constructing a metal–ligand polymeric nanogel with SOD and peroxidase (POD) activities; upon coupling with a fluorescent dye, it facilitated efficient ROS-responsive biofluorescence imaging in tumor-bearing mice through a cascade reaction converting excess superoxide radicals to H_2_O_2_ at the tumor site (Figure 15b).^204^ Wang’s group extensively researched enzyme-integrated nanogels (SOD, CAT) to generate ultrasound-sensitive oxygen bubbles via cascade enzymatic reactions at tumor sites, improving ultrasound imaging performance (Figure 15c).^205^ Additionally, reports detail the coupling of coumarin (Cou) dyes with short peptides, initiating ALP-catalyzed self-assembly into luminescent supramolecular nanofibers (LSN-pYD) that specifically distinguish cancerous tissues from normal tissues, functioning as diagnostic tools for early-stage tumors.^206^ Within an ALP-integrated hydrogel system, Xu and colleagues designed a fluorescein isothiocyanate (FITC)-based precursor (Fmoc-KFFyP) that undergoes ALP-triggered dephosphorylation, forming an amphiphilic gelator that self-assembles into a fluorescent enzyme-integrated hydrogel, demonstrating a sol–gel transition process corresponding to a fluorescence switch. This system was employed for quantitative ALP detection in living cells both in vitro and in vivo.^49,51,207,208^

**Figure 15:**
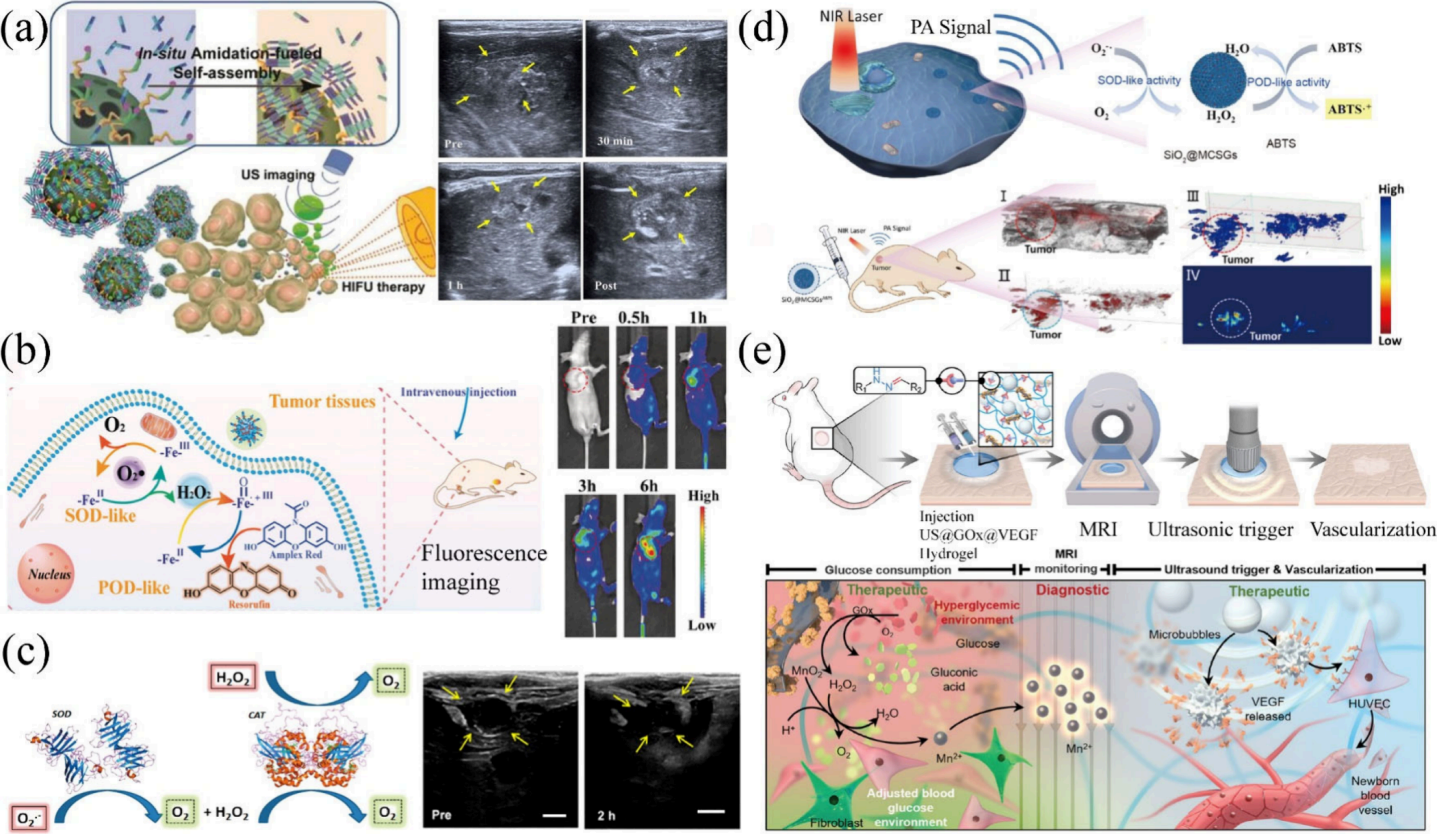
Enzyme-integrated hydrogels for bioimaging. (a) Mesoporous silica nanoparticle (MSN)-based ternary inorganic–supramolecular–polymeric hydrogels synthesized via amidation-fueled self-assembly and enzymatic post-cross-linking for ultrasound theranostic. Reproduced from ref 203. Copyright 2019 American Chemical Society. (b) Metal cross-linked polymeric nanogels (MPGs) prepared using atom transfer radical polymerization (ATRP) for ROS-responsive biofluorescence imaging. Reproduced with permission from ref 204. Copyright 2020 Wiley-VCH. (c) Enzyme-integrated nanogels that employ cascade enzyme reactions triggered by reactive oxygen species at the tumor site to generate ultrasound-sensitive oxygen bubbles, enhancing tumor-responsive ultrasound imaging performance. Reproduced with permission from ref 205. Copyright 2016 Royal Society of Chemistry. (d) Dilysine-liganded iron-functionalized peptide gels that react with modified silica by in situ amidation to self-assemble into SiO_2_-MCSG microgels with tandem SOD and POD catalytic properties, enabling efficient tumor photosensitive imaging after ABTS loading. Reproduced with permission from ref 210. Copyright 2021 Science China Press and Springer Nature. (e) Hydrogel patch loaded with enzymes and nanobubbles, enabling the monitoring of Mn^2+^ and glucose via MRI and promoting wound healing. Reproduced with permission from ref 213. Copyright 2023 Wiley-VCH.

Beyond ultrasound and fluorescence imaging, enzyme-integrated nanogels hold significant importance in photoacoustic imaging because of their responsiveness to external stimuli such as temperature and pH, multifunctional surface modifications, high drug and contrast agent loading capacity, and prolonged blood circulation time.^209^ Xia et al. designed the peptide-coordinated iron-functionalized monomer NapFFE-Fe­(Lys)_2_, based on the amino acid sequence Nap-Phe-Phe-Glu, using carboxyl-functionalized silica nanoparticles as the core.^210^ Inducing protonation through an amide reaction led to monomer self-assembly on the silica nanoparticles surface, creating a layer of iron–peptide nanogel (SiO_2_@MCSGs) with SOD and POD activities. Upon loading with the substrate ABTS, this nanogel catalyzed the conversion of superoxide anions to hydrogen peroxide at the tumor site, oxidizing ABTS and enabling efficient photoacoustic imaging of the tumor (Figure 15d).^210^ Furthermore, nanogel-integrated enzyme systems enhance magnetic resonance imaging (MRI) by stimulating the interaction of functional species with proximal water, which improves *T*_1_ and *T*_2_ relaxation and results in optimal imaging statistics,^211^ offering an excellent diagnostic tool for ultrasound and MRI techniques. For example, a hybrid hydrogel coloaded with iron oxide and manganese oxide was created via laccase-initiated assembly of superparamagnetic iron oxide particles into polysaccharide nanoclusters and served as a pH-responsive MRI contrast agent.^212^ Moreover, Han et al. developed an injectable self-healing HA hydrogel via the Schiff base reaction. This hydrogel was loaded with nanozyme (GOx-MnO_2_) and nanobubbles containing vascular endothelial growth factor (VEGF), thereby creating a system integrating both diagnostic and therapeutic processes.^213^ The loaded nanozyme rapidly consumes glucose in the microenvironment, producing H_2_O_2_, which in turn reacts with manganese dioxide to generate Mn^2+^, thereby enhancing MRI performance (Figure 15e).^213^ The hydrogel wound patch enabled MRI monitoring of Mn^2+^ levels from 0.5 to 7.8 × 10^–3^ M and glucose levels from 100 × 10^–3^ to 3 × 10^–3^ M, offering a novel approach for integrated diagnosis and treatment and presenting a new concept for chronic wound healing.

Enzyme-integrated macrohydrogels are employed as biosensors for monitoring biological signals due to their highly hydrophilic three-dimensional networks and unique physicochemical properties, including flexibility, resilience, biodegradability, biocompatibility, distinctive enzyme properties, and responsiveness to microenvironmental stimuli. In contrast, enzyme-integrated microgels not only benefit from their small size for better body penetration and prolonged bloodstream circulation but also possess favorable channels for substrate molecule diffusion, exhibit enhanced backscattering signals and visualization effects, and demonstrate responsiveness to the in vivo microenvironment. Consequently, enzyme-integrated microgels are ideal candidates for nanomedical imaging and diagnostic studies, and the development of multimodal biosensor systems based on these microgels lays the foundation for future integrated enzyme-based diagnostic and therapeutic systems.

### 3.2 Enzyme-Integrated Hydrogels for Biotherapeutic Applications

Enzyme-integrated hydrogels represent an emerging biotherapeutic platform. Enzyme therapy employs bioengineering techniques to synthesize biological enzyme products for treating malignant tumors by restoring internal environment balance and modulating cellular metabolism. Numerous studies have documented the use of single-enzyme catalysis targeting tumor metabolites in adjuvant therapy, primarily involving metalloproteases,^57,214,215^ hydrolases,^216,217^ and oxidases to locally elevate reactive oxygen species levels.^218–220^ However, reports on direct metabolic intervention in tumors using multienzyme systems—which offer superior catalytic efficiency, selectivity, and mild reaction conditions—remain relatively scarce. Furthermore, while various nanomaterials have been developed to disrupt cellular redox homeostasis and induce apoptosis by responsively modulating aerobic metabolism, their therapeutic efficacy is often limited by factors such as ROS instability within the tumor microenvironment and inadequate sustainability of exogenous stimuli. To address these challenges, researchers have adopted enzyme-integrated hydrogels to intervene in cellular aerobic metabolism through enzymatic oxidation cascades, establishing a tumor biotherapy strategy centered on redox homeostasis.^221,222^ For instance, Xu et al. described a glycopolymer functionalized with phenolic hydroxyl groups that undergoes gelation via a cascade reaction mediated by GOx and horseradish peroxidase, forming a hydrogel system (Figure 16a).^132^ This system achieved high-efficiency encapsulation of mannose and continuously disrupted the tumor energy supply through efficient glucose metabolism via cascade catalysis, significantly enhancing anti-tumor efficacy. Expanding on enzymatic ROS regulation, Wu et al. engineered an active nanogel system incorporating superoxide dismutase and chloroperoxidase self-assembled around iron oxide nanoparticles. This system effectively converts ROS into hypochlorous acid (HOCl) and cytotoxic singlet oxygen (^1^O_2_) within tumors, achieving potential tumor suppression.^137^ Additionally, a chemodynamic immunotherapy strategy utilizes GOx–ferrocene (Fc) cascade, where DNA programmability controls the spatial arrangement between catalysts to maximize glucose conversion into hydroxyl radicals, thereby promoting immunogenic cell death (Figure 16b).^223^ Recent advances increasingly integrate enzymes with physical stimuli to enhance tumor treatment efficiency.^224,225^ For example, the magnetic-responsive nanogel MNP-CPO@Nanogels disrupt tumor oxygen homeostasis upon alternating magnetic field stimulation. This induces H_2_O_2_ production, which chloroperoxidase converts into singlet oxygen, elevating ROS levels for effective tumor therapy.

**Figure 16:**
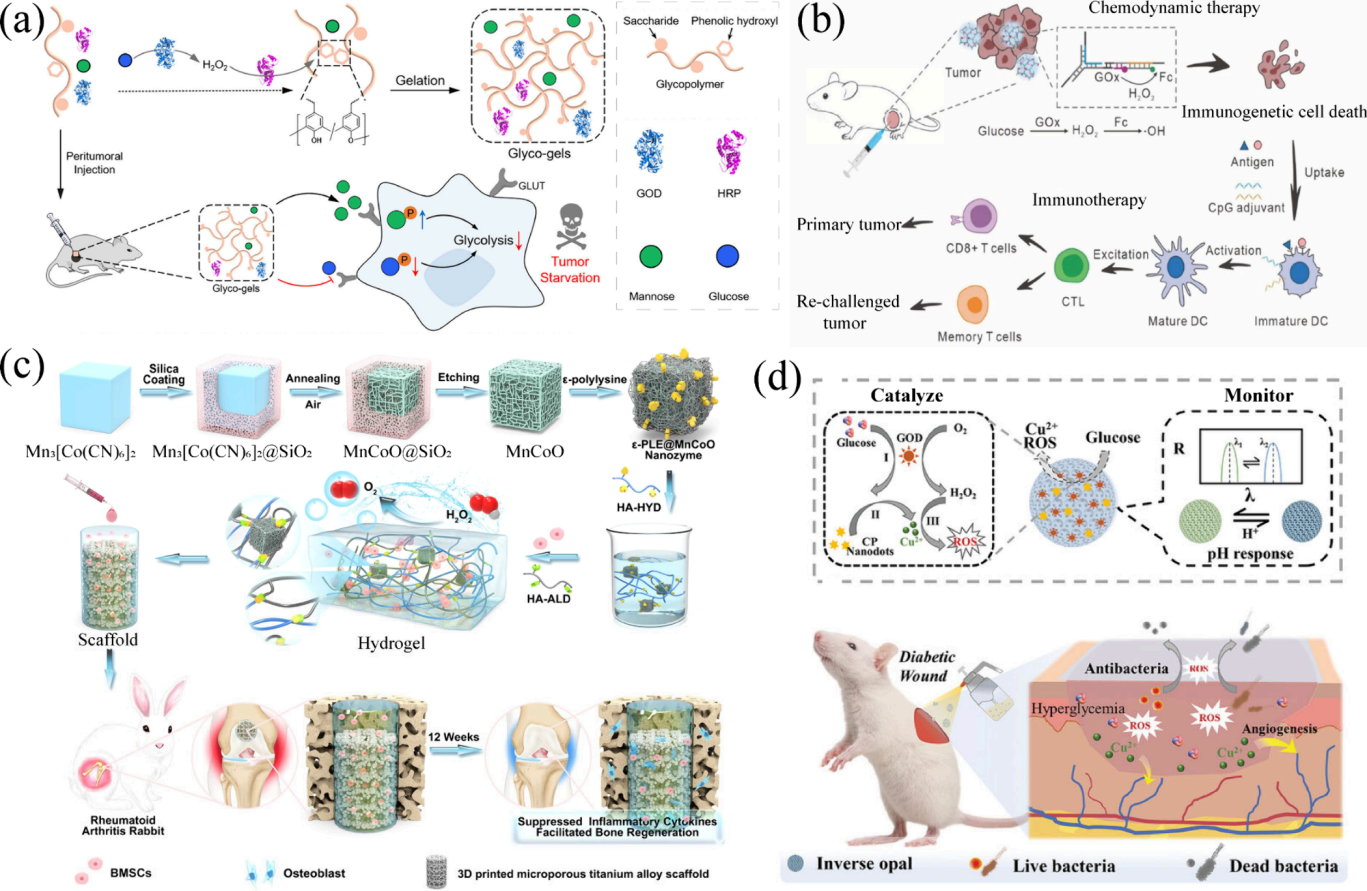
Enzyme-integrated hydrogels for oncology and regenerative medicine. (a) Cascade catalysis of GOx and HRP enables the synthesis of glycopolymer-based hydrogels, effectively suppressing tumor growth by releasing mannose and consuming glucose. Reproduced from ref 132. Copyright 2023 American Chemical Society. (b) DNA adjuvant hydrogels incorporating CpG motifs (CpG ODNs) were fabricated to establish a GOx–ferrocene (Fc) cascade for tumor chemodynamic immunotherapy. Reproduced from ref 223. CC BY 4.0. (c) A nanozyme-based hydrogel, developed as a H_2_O_2_-driven oxygenerator, aims to improve the osseointegration of prosthetic interfaces in rheumatoid arthritis treatment. Reproduced from ref 226. CC BY 4.0. (d) Construction of a hydrogel via photopolymerization using GOx and copper peroxide-containing inverted opal microparticles that employs a cascade reaction to treat diabetic wound infection. Reproduced from ref 234. CC BY 4.0.

Leveraging the inherent safety, efficiency, and specificity of enzyme therapy, macroscopic enzyme-integrated hydrogels offer promising strategies for regenerative medicine, including cartilage repair, tissue regeneration, and wound hemostasis. Utilizing wound glucose as a substrate, one study developed a GOx/glycine ferrous system (GOx/Fe­[Gly]) for ultrafast gelation of acryloyl-modified chondroitin sulfate (acryloylated-CS) and *N*,*N*-dimethylacrylamide (DMAA), creating injectable CS-DMAA hydrogels as an injectable tissue filler for cartilage repair.^150^ Similarly, Zhao et al. engineered a nanozyme-reinforced hydrogel delivering H_2_O_2_-driven oxygenerators to modulate stem cell behavior (Figure 16c).^226^ In response to hypoxia and oxidative conditions in rheumatoid arthritis (RA) synovium, this system decomposes endogenous H_2_O_2_ to generate oxygen, alleviating the pathological microenvironment, while inhibiting inflammatory cytokines and improving prosthetic interface osseointegration.

Diabetic wound healing poses significant challenges due to persistent bacterial infection,^227,228^ hypoxia,^229^ hyperglycemia,^230,231^ and oxidative stress.^232,233^ Wang et al. designed a cascade-catalyzed inverse opal hydrogel with immobilized GOx and chitosan nanodots.^234^ Upon application, the hyperglycemic environment fuels GOx-mediated H_2_O_2_ generation, while the acidic wound environment triggers nanodot degradation to further catalyze antimicrobial ROS production, synergistically accelerating healing (Figure 16d).^234^ Another approach cascades urate oxidase (UOX) and HRP to construct a secondary gel network while catalyzing uric acid oxidation into therapeutic allantoin for enhanced skin repair.^235^ Researchers have also developed hydrogels incoporating MoS_2_ nanosheets and bovine serum albumin-modified gold nanoparticles (MoS_2_@Au@BSA NS) with multiple mimetic enzyme activities.^236^ Here, glucose oxidation by Au generates H_2_O_2_, while the peroxidase-like activity converts H_2_O_2_ into bactericidal hydroxyl radicals, accelerating wound healing through combined glucose depletion and ROS-mediated clearance.

### 3.3 Potential Applications of Enzyme-Integrated Hydrogels Across Emerging Fields

Enzyme-integrated hydrogels demonstrate significant potential beyond their established roles in biological diagnostics and therapeutics, extending their applications to diverse applications across multiple fields. In three-dimensional and stem cell culture systems, Deller et al. successfully immobilized a thrombin–polymeric surfactant complex onto mesenchymal stem cell membranes. This configuration enables cell-surface-bound thrombin to catalyze localized fibrin hydrogel formation, effectively encapsulating cells while preserving their stemness to support both proliferation and differentiation.^237^ Complementary studies have further demonstrated hydrogel formation around cells through HRP conjugation to membrane-bound molecules,^238,239^ highlighting the broader utility of enzyme-integrated systems for advanced 3D culture and organoid engineering.

The functional versatility of these systems is further amplified through strategic material integration (Figure 17). Combining stimuli-responsive materials with enzymes enables fabrication of smart hydrogels for high-sensitivity biosensing applications. Similarly, nanomaterial-incorporated platforms leverage both enzymatic mimicry and intrinsic properties (e.g., photothermal/electromagnetic characteristics) to create multifunctional hydrogels suitable for remote sensing and electronic devices. Moreover, enzyme integration with organic components facilitates hydrogel platforms applicable to environment remediation (e.g., pollutant degradation), energy technologies (storage/conversion systems), and intelligent soft robotics, demonstrating considerable cross-disciplinary potential.

**Figure 17:**
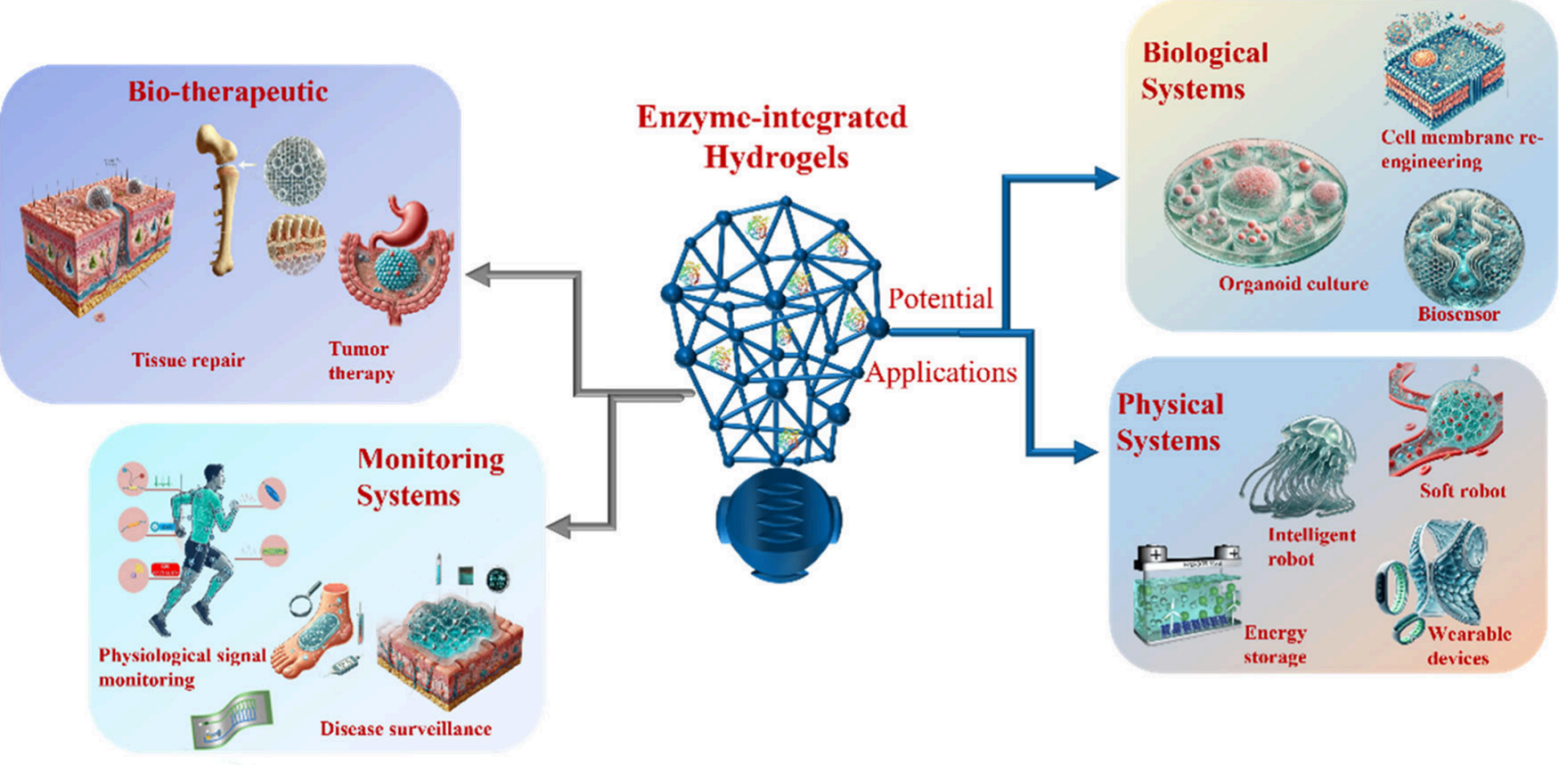
Schematic illustration of enzyme-integrated hydrogel applications across various fields.

## 4 Concluding Remarks

This review systematically examines fabrication strategies for enzyme-integrated hydrogels, categorizing them into physical methods (e.g., self-assembly, electrostatic interactions, direct encapsulation) and enzyme-mediated approaches (e.g., single-enzyme, cascade-enzyme, and nanozyme systems). It further highlights their innovative applications in disease diagnosis (bioimaging and biomarker monitoring) and therapy (cancer treatment and tissue regeneration).

Physical integration techniques, such as electrostatic adsorption and direct entrapment, offer advantages such as simplicity, mild reaction conditions, and operational efficiency. The inherent reversibility of non-covalent interactions also facilitates responsive system behavior. However, these methods suffer from limitations in encapsulation efficiency and selective enzyme–matrix compatibility. In contrast, enzymatic polymerization/cross-linking overcomes critical bottlenecks of conventional methods (e.g., photopolymerization, thermal polymerization)—such as oxygen inhibition and poor biocompatibility—by leveraging mild reaction conditions, tunable substrate selectivity, and high product purity. The complementary strengths and limitations of these strategies are comparatively summarized in Table 2, highlighting their synergistic potential in biomedical applications.

**Table 2:** Comparative Analysis of Enzyme Integration Techniques in Hydrogels for Biomedical Applications

strategy	method	representative enzymes	advantages	limitations	biomedical applications	refs
physical methods	self-assembly	ALP, peptide-specific enzymes	mild conditions; preserves native enzyme conformation	limited loading capacity; moderate stability; risk of enzyme leakage; specific enzyme structural requirements	wound dressings; controlled drug release	^240^,^241^
	electrostatic adsorption	charged enzymes (e.g., lysozyme, GOx)	simple, rapid, and reversible binding	sensitive to pH/ionic strength; leakage risk; potential activity modulation by local charges; moderate loading/stability	antimicrobial coatings; biosensors	^60^,^242^
	direct encapsulation	broad range	simple; high loading capacity	significant enzyme leakage; poor control over enzyme localization/distribution; limited long-term stability	microenvironment modulation; metabolic disease therapy	^243^,^244^
chemical methods	single-enzyme-mediated systems	TYR, laccase, etc.	minimal leakage; enhanced stability; improved spatial control	risk of activity loss via chemical modification; may hinder substrate diffusion; biocompatibility concerns	in vitro diagnostics; biocatalysis	^245^,^246^
	cascade-enzyme-mediated systems	GOx/HRP	enables complex multistep reactions; enhances efficiency/specificity; reduces intermediate diffusion limits	differential immobilization efficiency/activity retention; complex optimization; spatial coordination challenges	glucose monitoring; cancer therapy (starvation/ROS generation)	^247^,^248^
	nanozyme/enzyme-mimic systems	nanozymes (Fe_3_O_4_, CeO_2_, MOFs), organocatalysts	high stability; tunable design; cost-effective; high environmental tolerance	generally lower activity/specificity vs natural enzymes; requires careful biocompatibility assessment; risk of off-target reactions	antibacterial/antioxidant therapies	^249^,^250^
	integrated physico-chemical systems	GOx, CAT	precise spatial control; improved stability	requires optimization of synergistic effects; biocompatibility concerns	wearable biosensors; tissue engineering	^251^,^252^
alternative methods	genetic fusion proteins	recombinant luciferase	precise spatial control; improved stability	requires advanced biotechnological expertise	molecular imaging; gene therapy	^253^,^254^

The integration of enzymes into hydrogel networks spans a continuum of structural scales, encompassing discrete microgels and continuous macrogel frameworks. For microgels, enzyme-catalyzed polymerization exhibits distinct advantages, including precise control over microsphere size, enhanced targeting specificity, prolonged circulation time, and facile integration with imaging agents (e.g., fluorescent dyes and MRI contrast agents), positioning them as powerful tools for tumor imaging and targeted therapy. In macrogel systems, enzyme-integrated hydrogels furnish an ideal cell-growth microenvironment due to their high hydrophilicity, biomimetic properties, tunable elastic modulus, and 3D porous architecture. These features promote efficient nutrient–waste exchange and accelerate tissue repair. Critically, such hydrogels can remodel pathological microenvironments via enzymatic reactions, enabling sustained regulation of reactive oxygen species (ROS) for diverse therapeutic needs.

Although immobilization enhances enzyme stability, activity attenuation in complex physiological environments remains a core challenge for clinical translation. Current systems rely heavily on hydrolases and oxidoreductases, whose inherent thermosensitivity and pH dependence lead to inactivation in pathological settings. Recent advances address this limitation, as exemplified by GOx immobilized within a polydopamine-coated ZIF-8 composite (GOx@ZIF-8/PDA), which retains over 90% activity after five reuse cycles—significantly surpassing free GOx. This stability originates from synergistic PDA adhesion and ZIF-8 confinement, which collectively stabilize the enzyme conformation.^255^ Similarly, enzyme-imprinted MOFs create size/shape-tailored cavities that yield immobilized enzymes exhibiting 16.7-fold higher activity and 14.1-fold greater catalytic efficiency (*K*_cat_/*K*_m_) compared to free enzymes. These improvements are achieved through confined folding dynamics and cofactor coordination within the imprinted chambers, which reshape active sites and precisely regulate the enzymatic microenvironment.^256^

In cascade system design, in vitro platforms benefit from low flux barriers and operational simplicity but face high costs, exemplified by multistep purification processes for GOx/Hb nanoflowers that significantly increase production expenses. Conversely, intracellular cascades—though cost-effective—grapple with reduced catalytic efficiency, metabolic interference, and intermediate toxicity. For example, Mn^2+^–lysozyme hydrogels risk releasing neurotoxic Mn species in acidic wounds despite activating cGAS-STING pathways.^257^ Advanced techniques like microfluidics (e.g., two-photon lithography fabrication of cell-laden microspheres at ≤6 μm resolution) improve uniformity but confront scalability and equipment-cost hurdles.^258^

Immunogenicity control is paramount for long-term safety. Exogenous enzymes (e.g., HRP) may trigger IgE-mediated hypersensitivity,^259^ while nanozymes (e.g., ceria, MXene) risk inorganic bioaccumulation. Even native enzymes can expose novel epitopes upon conformational changes during integration,^260^ necessitating robust immunogenicity screening. Additional translational barriers include batch-to-batch variability in enzyme activity, inadequate long-term safety data, and outdated evaluation standards.

Future efforts must prioritize developing smart systems that utilize stimuli-responsive materials and enable in vivo self-assembly for fabricating enzyme-integrated hydrogels such as via bioorthogonal enzyme-catalyzed hydrogelation. Researchers should advance computational and AI-driven design methodologies, including combining molecular dynamics simulations with machine learning algorithms to predict enzyme–carrier interactions. Establishing standardized evaluation protocols is essential to systematically assess critical parameters like enzyme activity retention, release kinetics, and metabolite toxicity. Implementing modular manufacturing processes will be crucial for achieving scalable production. Finally, conducting comprehensive long-term safety assessments represents a fundamental requirement for clinical translation.

In conclusion, enzyme-integrated hydrogels represent a transformative frontier in biomaterials, offering immense potential for diagnosis, therapy, and regenerative medicine. Through enzyme-catalyzed polymerization and physical integration, these systems achieve advanced functions, including microenvironment remodeling, targeted delivery, and stimuli-responsiveness. However, clinical translation hinges on resolving enzyme stability, cascade cost, and immunogenicity. Overcoming these demands requires interdisciplinary convergence of enzyme engineering, materials science, artificial intelligence, and advanced manufacturing. Advances in in vivo self-assembly, biomimetic catalysis, and rigorous safety frameworks promise to bridge the gap from “proof of concept” to clinical ubiquity, ultimately enabling personalized precision medicine.
